# Full-Length Synaptonemal Complex Grows Continuously during Meiotic Prophase in Budding Yeast

**DOI:** 10.1371/journal.pgen.1002993

**Published:** 2012-10-11

**Authors:** Karen Voelkel-Meiman, Sarah S. Moustafa, Philippe Lefrançois, Anne M. Villeneuve, Amy J. MacQueen

**Affiliations:** 1 Department of Molecular Biology and Biochemistry, Wesleyan University, Middletown, Connecticut, United States of America; 2 Department of Molecular, Cellular, and Developmental Biology, Yale University, New Haven, Connecticut, United States of America; 3 Department of Developmental Biology, Stanford University, Stanford, California, United States of America; Stowers Institute for Medical Research, United States of America

## Abstract

The synaptonemal complex (SC) links two meiotic prophase chromosomal events: homolog pairing and crossover recombination. SC formation involves the multimeric assembly of coiled-coil proteins (Zip1 in budding yeast) at the interface of aligned homologous chromosomes. However, SC assembly is indifferent to homology and thus is normally regulated such that it occurs only subsequent to homology recognition. Assembled SC structurally interfaces with and influences the level and distribution of interhomolog crossover recombination events. Despite its involvement in dynamic chromosome behaviors such as homolog pairing and recombination, the extent to which SC, once installed, acts as an irreversible tether or maintains the capacity to remodel is not clear. Experiments presented here reveal insight into the dynamics of the full-length SC in budding yeast meiotic cells. We demonstrate that Zip1 continually incorporates into previously assembled synaptonemal complex during meiotic prophase. Moreover, post-synapsis Zip1 incorporation is sufficient to rescue the sporulation defect triggered by SCs built with a mutant version of Zip1, Zip1-4LA. Post-synapsis Zip1 incorporation occurs initially with a non-uniform spatial distribution, predominantly associated with Zip3, a component of the synapsis initiation complex that is presumed to mark a subset of crossover sites. A non-uniform dynamic architecture of the SC is observed independently of (i) synapsis initiation components, (ii) the Pch2 and Pph3 proteins that have been linked to Zip1 regulation, and (iii) the presence of a homolog. Finally, the rate of SC assembly and SC central region size increase in proportion to Zip1 copy number; this and other observations suggest that Zip1 does not exit the SC structure to the same extent that it enters. Our observations suggest that, after full-length assembly, SC central region exhibits little global turnover but maintains differential assembly dynamics at sites whose distribution is patterned by a recombination landscape.

## Author Summary

Sexually reproducing parents use meiosis to generate specialized cells in which chromosome sets are reduced from two to one. Accurate chromosome reduction relies on the prior establishment of pair-wise associations between homologous chromosomes (homologs); maintenance of paired associations typically occurs via interhomolog crossover recombination events. The proteinaceous, structurally conserved synaptonemal complex (SC) assembles along the full length of aligned homolog axes. The SC is the context in which crossover recombination events mature, and it influences both the level and distribution of crossover events between homologs. However it is not clear whether the SC maintains the capacity to remodel, for example to structurally accommodate recombination events. We explore the dynamics of budding yeast SC and find that full-length SCs exhibit ongoing subunit incorporation but little subunit turnover during a meiotic cell cycle arrest, thus SC grows over time. Interestingly, initial subunit incorporation into full-length SCs occurs predominantly at or adjacent to Zip3 foci, a presumed marker of crossover sites. Our observations suggest that budding yeast SC continues to assemble during a steady state and that, while it may have little capacity for global turnover after installation, the SC maintains differential assembly dynamics at recombination-associated perturbations in the meiotic axis.

## Introduction

The critical events that ensure a precise reduction in chromosome ploidy at the first meiotic division occur during meiotic prophase [1], [2]. Chromosomes are typically unpaired as nuclei enter meiosis, but by late prophase have established connections with their homologous partners, which ultimately allow such partners to disjoin specifically from one another at the first meiotic division (and segregate to separate daughter nuclei). Thus, a key accomplishment of meiotic prophase is the formation of stable partnerships between homologous chromosomes.

The process that drives homolog pairing can be divided into two major steps: initiation and reinforcement. The molecular mechanism that mediates initial pairing between partner chromosomes is still unclear, but must involve the capacity to recognize homology and productively link this recognition to reinforcement of a paired association between two chromosomes. For meiotic nuclei in most organisms, homolog associations are stabilized for the long term via a crossover recombination event. DNA double-strand breaks (DSBs) are deliberately induced and undergo regulated repair during meiotic prophase; the fraction of double-strand breaks that are repaired to a crossover outcome involving the homolog's (nonsister) chromatid ensure that homologous partner chromosomes are linked, so long as sister cohesion remains intact.

A physical and functional link between homology recognition and stable maintenance of chromosomal partnerships is the synaptonemal complex (SC). SC formation (synapsis) involves the multimeric assembly of coiled-coil containing proteins; the coiled-coil containing proteins that establish the SC central region interact with themselves and with chromosome axis proteins associated with each homolog in order to ultimately generate an elaborate protein lattice at the interface of lengthwise-aligned chromosomes [2], [3]. SC links initial homolog pairing with pairing maintenance by virtue of the fact that SC assembly is normally regulated such that it occurs only subsequent to homology recognition between chromosomes, whereas a fully assembled SC may be required for a normal number and distribution of crossover recombination events (which will in turn ensure the persistence of homologous associations after SC disassembly and until chromosome segregation on the meiosis I spindle) [2]–[8].

Zip1 is a primary structural component of the SC central region in budding yeast [9]–[11]. Like most SC central region proteins identified to date, Zip1 contains an extensive coiled-coil domain flanked by globular ends, and is predicted to form dimers. The structure of SC central region proteins resembles that of intermediate filament subunits and suggests a capacity to self-assemble [3], [12]. Elegant immuno-electron microscopy experiments in budding yeast demonstrated that Zip1 subunits interact with one another near their amino termini, and interact with chromosome cores at their carboxyl terminal ends [9]. Additional proteins that do not share structural similarity to typical transverse filament proteins may also contribute to establishing SC central region. For example, the Small Ubiquitin-like MOdifier protein, SUMO, has been implicated in SC central region assembly on the basis of a dependence on central region proteins for its localization to SC [12]–[15].

The Synapsis Initiation Complex (SIC) proteins Zip2, Zip3 and Zip4 promote SC central region assembly from distinct synapsis initiation sites along the length of the chromosome [16]–[18]. A set of synapsis initiation sites corresponds to centromeres, while the remainder are presumed, based on the number and distribution of SIC foci along chromosome arms, to correspond to crossover recombination sites [19]. Interestingly, SIC proteins remain localized as discrete foci along chromosomes even after full-length SC has been deposited [16]–[18], raising the question of whether SICs have a later role in crossover recombination or SC maintenance at discrete sites, after their initial role in SC assembly.

The existence of multiple layers of regulation that prevent inappropriate synapsis initiation suggests that SC may act as an irreversible tether between chromosomes [5]. Initial SC assembly interfaces closely with the homolog pairing process: Observations in multiple organisms of SC protein aggregation (polycomplex formation) in pairing-defective meiotic mutants indicate that cells normally regulate SC subunits such that their assembly on chromatin is contingent on homology verification [2], [6], [20], and this notion has been borne out by the identification of checkpoint-like pathways that prevent SC assembly when homologous pairing fails [5], [21], [22]. However, the possibility of inappropriate initiations or interlocks between partially synapsed chromosome pairs raises the question of whether the SC central region maintains a capacity to remodel. Furthermore, assembled SC is the context within which meiotic recombination events mature, and analyses of SC-deficient meiotic mutants suggest that the SC functionally interfaces with at least a subset of recombination events [17], [18], [23], [24]. Whether the dynamics or composition of SC central region is altered at Spo11-mediated recombination sites is not known.

Here we investigate the dynamics of the fully assembled SC in budding yeast meiotic cells that are arrested at the pachytene stage of meiotic prophase (when chromosomes are fully synapsed). Multimeric protein structures that exhibit both ongoing incorporation and ongoing exit (“treadmilling”) might be rapidly disassembled by lowering the “on-rate” in a local region; perhaps the budding yeast SC structure exhibits such dynamics that would enable it to disassemble quickly. On the contrary, we demonstrate that Zip1 continually incorporates into full-length synaptonemal complex and that, globally, Zip1 does not exit to the same extent as it enters the SC structure in pachytene-arrested cells. The rate of SC assembly and maximum size of SC central region increases in direct proportion to Zip1 copy number. Our observations suggest that the budding yeast SC structure behaves more like a “tether” than a “treadmill” during pachytene arrest. Interestingly, fully assembled SCs exhibit a non-uniformity in SC assembly dynamics such that initial post-synapsis Zip1 incorporation is favored in the vicinity of recombination events.

## Results

### Full-Length Synaptonemal Complex Exhibits Ongoing Incorporation of Zip1

To explore the dynamics of the fully formed SC, we created a strain with inducible expression of the SC central region subunit, Zip1 (or a tagged version, Zip1-GFP, a kind gift of D. Kaback [25]), using the estrogen-regulated Gal4-ER transcription factor in *trans* [26], [27]. In the absence of β-estradiol, chromosome spreads from meiotic cells in which *ZIP1* or *ZIP1-GFP* is solely under *GAL1* promoter control exhibited a *zip1* null phenotype: immunostaining of meiotic prophase chromosomes demonstrated that little to no Zip1 localizes to chromosomes in uninduced nuclei, and chromosomal axes often exhibited axial associations (intermittent points of contact presumed to be crossover recombination events, flanked by regions of abnormally loose axial alignment between homologs) (Figure S1). Moreover, in the absence of β-estradiol, the sporulation efficiency and spore viability of our “inducible-SC” strain phenocopied the *zip1* null (Figure S1).

On the other hand, when sporulated in the presence of β-estradiol, cells carrying an inducible *ZIP1* allele exhibited Zip1 or Zip1-GFP at the interface of aligned, homologous chromosome axes, and the sporulation efficiency as well as the viability of the spore products of these meiotic cells was rescued to wild-type levels (Figure S1). These data indicate that the estrogen-inducible Zip1 can functionally substitute for endogenous Zip1. Interestingly, when this strain was made homozygous for an *ndt80* mutation, which arrests otherwise wild type cells at late prophase with fully synapsed chromosomes [28], we observed that Zip1 can assemble *de novo* at the interface of homologous axes that have progressed to late prophase without SC (Figure S2).

**Figure 1:**
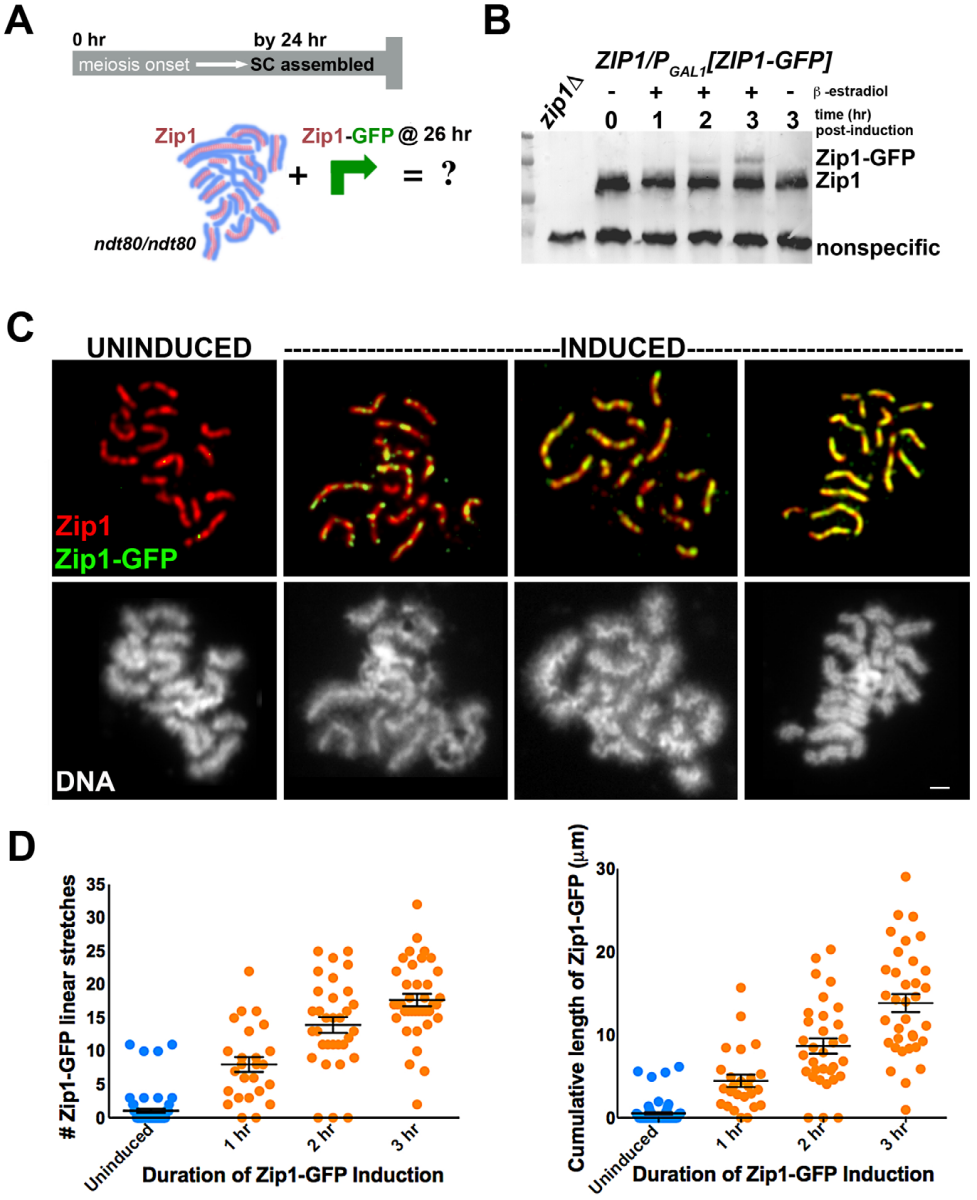
Zip1-GFP incorporates into previously established, full-length SC. Cartoon in (A) outlines the induction experiment designed to explore the dynamics of the full-length SC structure using K39. Most *ndt80* mutant meiotic nuclei exhibit full-length SC by 24 hours of sporulation; *ndt80* meiotic cells are induced to express *ZIP1-GFP* at 26 hours of sporulation. Western blot in (B) has been probed with an anti-Zip1 antibody, which detects both untagged and tagged Zip1 species, labeled at right (although relative affinity of this antibody for either version of Zip1 is unknown). (C) Representative images of surface-spread nuclei from uninduced or induced cells. Staining for Zip1 (and/or Zip1-GFP) (red) and Zip1-GFP (green) is shown in top panels while corresponding DAPI-stained DNA (white) is pictured below). Zip1-GFP is barely detectable in most uninduced nuclei but exhibits dotty to linear decoration of SCs in induced nuclei. Scale, 1 µm. Scatterplots in (D) display the number of Zip1-GFP stretches that are 0.35 µm or longer associated with full-length SCs per nucleus (left) or the cumulative length of Zip1-GFP per nucleus (right) after various periods of *ZIP1-GFP* induction. Circles indicate values for individual nuclei; horizontal lines and error bars represent the mean and standard error of the mean, respectively.

To explore the dynamics of the full-length SC in budding yeast, we next created a second *ndt80* diploid strain, K39, in which one *ZIP1* locus encodes a tagged version of *ZIP1* (*ZIP1-GFP*) under *GAL1* promoter control, while the other *ZIP1* locus is unmodified. After 24 hours of sporulation, (82%, n = 148) of K39 meiotic nuclei exhibited full length SCs comprised of untagged Zip1 (as judged by the near absence of GFP on spread meiotic chromosomes). We used this K39 strain to ask: Will new Zip1 subunits continue to incorporate into pre-existing full-length SC structures, or will such superfluous Zip1 subunits become sequestered into a polycomplex structure? (Figure 1A). K39 meiotic cells that had been sporulating for 26 hours (and thus predominantly containing full-length SC) were exposed to β-estradiol to induce the expression of Zip1-GFP (Figure 1B). Surface spread nuclei from uninduced and induced meiotic cells harvested 1, 2 or 3 hours after β-estradiol addition were immunostained with anti-GFP and anti-Zip1 antisera to monitor the distribution of newly induced Zip1-GFP subunits, relative to previously assembled SC.

Our induction experiment revealed that new Zip1-GFP readily incorporates into previously established, full length SCs instead of forming polycomplex (Figure 1C, 1D). 96% (n = 25) of cells harvested after 1 hour of incubation with β-estradiol exhibited Zip1-GFP incorporation along the length of fully formed SC. Of these nuclei, none exhibited polycomplex. Longer incubation in β-estradiol correlates with increasing levels of Zip1-GFP incorporation: over half of nuclei monitored after 2 hours of induction exhibited Zip1-GFP completely coincident with unlabeled Zip1 along extensive lengths of SC (Figure 1D). Uninduced control nuclei exhibited rare GFP staining, resulting from either a low level of constitutive expression allowed by the inducible promoter system in some cells, or from nonspecific background staining.

When we examined Zip1-GFP induction in meiotic mutants, such as *zip3*, that display polycomplex in addition to SC stretches, induced nuclei always exhibited Zip1-GFP incorporation into polycomplex (as well as into SC stretches, see below).

### Post-Synapsis Incorporation of Wild-Type Zip1 Rescues the Meiotic Arrest Associated with Zip1-4LA SC

To explore whether the newly induced Zip1-GFP that decorates pre-established SCs is functionally incorporated, we took advantage of a previously characterized *zip1* allele, *zip1-4LA* [29]. While *zip1* null mutant cells (of the BR1919 genetic background) fail to build SC but do sporulate at reduced levels, *zip1-4LA* homozygotes build full-length SC, exhibit a normal level of crossover recombination but altogether fail to make spores. Instead, *zip1-4LA* meiotic cells arrest late in prophase with fully synapsed chromosomes (thus *zip1-4LA* meiotic cells cytologically resemble *ndt80* meiotic cells at late prophase). Mitra and Roeder (2007) previously suggested that the pachytene arrest exhibited by *zip1-4LA* cells is triggered by an assembled (albeit defective) SC, based on their observation that *spo11 zip1-4LA* double mutants, in which Zip1-4LA is expressed but fails to assemble SC, sporulate to the same extent as *spo11* single mutants. We reasoned that if Zip1 subunits functionally incorporate into previously deposited, full-length SC, then post-synapsis expression of wild-type Zip1 (or Zip1-GFP) may be sufficient to “remodel” SCs initially built of Zip1-4LA protein and suppress the meiotic arrest associated with Zip1-4LA SCs.

**Figure 2:**
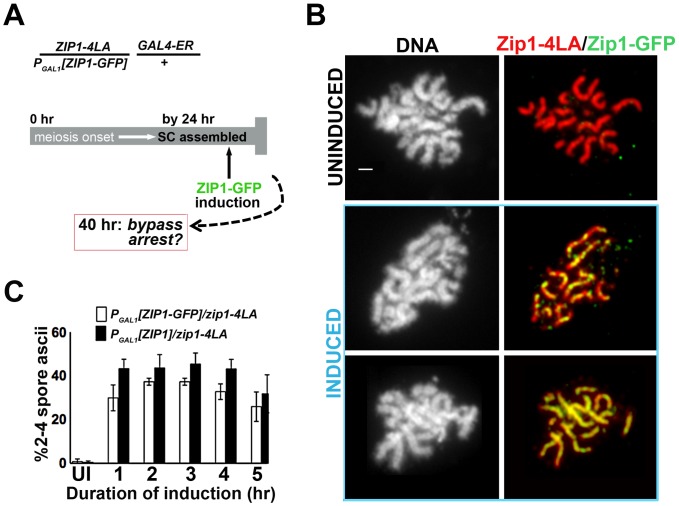
Post-synapsis incorporation of Zip1 or Zip1-GFP suppresses sporulation arrest triggered by SC built with Zip1-4LA. (A) Cartoon outlines the induction experiment carried out using K29 and K30. Meiotic cells expressing Zip1-4LA, instead of Zip1, arrest in late prophase with full-length SCs. At 24 hours of sporulation, most *zip1-4LA/P_GAL1_[ZIP1-GFP] GAL4-ER/+* meiotic prophase-arrested cells exhibit full-length SC. *ZIP1* or *ZIP1-GFP* expression was induced in such cells at 24 hours, and then either processed for chromosome spreads (at 1 hour post-induction) or scored for spore formation (at 16 hours post-induction). (B) Nuclei from uninduced (upper panels) and induced (two examples, lower panels) K30 cells, were surface-spread and labeled with DAPI (white), anti-Zip1 (red), anti-GFP (green) following 1 hour of *ZIP1-GFP* induction; lower panels show that SC built with Zip1-4LA readily incorporates induced Zip1-GFP. Scale, 1 µm. (C) Bar graph depicting quantitation of spore production by uninduced cells expressing only Zip1-4LA and following induction of Zip1 or Zip1-GFP. Cells containing *P_GAL1_[ZIP1]* or *P_GAL1_[ZIP1-GFP]* and *GAL4-ER* were exposed to β–estradiol for indicated time periods before washing in sporulation media; spore formation was assayed approximately 16 hours later. While uninduced cells produced less than 0.5% spores, cells exposed to induced Zip1 or Zip1-GFP after 24 hours of sporulation produced between 30–50% spores, which is near wild-type for this BR1919 strain background. 1000 meiotic cells were assayed for each strain at each time point; shown are data from three independent experiments.

To examine the possibility that new incorporation of Zip1 can remodel and alter the behavior of a full-length SC, we built a strain in which one chromosomal *ZIP1* locus carries *P_GAL1_[ZIP1]* or *P_GAL1_[ZIP1-GFP]* while the other chromosome contains the *zip1-4LA* allele under the endogenous *ZIP1* promoter (Figure 2A). After 24 hours in sporulation media, over 90% of meiotic nuclei from this strain exhibited late prophase chromosome morphology and full length SCs (built of Zip1-4LA protein), with little GFP visible in those meiotic nuclei carrying the *P_GAL1_ [ZIP1-GFP]* cassette (Figure 2B). Moreover, less than 1% of these cells eventually formed spores or spore-like structures after 40 hours in sporulation media (Figure 2C). When β-estradiol was added to the *P_GAL1_[ZIP1-GFP]* - containing strain at the 24 hour time point, Zip1-GFP was readily detected in full-length SCs after just one hour of induction (Figure 2B). Thus, SCs built of Zip1-4LA are capable of incorporating Zip1-GFP subunits. Moreover, cells from strains containing either the *ZIP1* or *ZIP1-GFP* inducible allele that were sporulated in the presence of β-estradiol exhibited near wild-type sporulation efficiency (Figure 2C). These results indicate that post-synapsis incorporation of Zip1 can functionally alter SC behavior.

### Sites of Initial Zip1 Incorporation into Previously Established SC Localize near a Synapsis Initiation Protein

Early Zip1-GFP incorporation into previously established, full-length SC exhibits a non-uniform pattern (Figure 1C, Figure 2B). Instead of a uniform appearance throughout the SC surface, discrete Zip1-GFP foci initially decorate the previously established SC. This pattern suggests the existence of discrete sites along the length of the SC where Zip1 incorporation is favored. As SC normally builds from multiple discrete sites along the length of chromosomes during its assembly, we asked whether such sites of initial Zip1-GFP entry exhibit characteristics of synapsis initiation sites.

**Figure 3:**
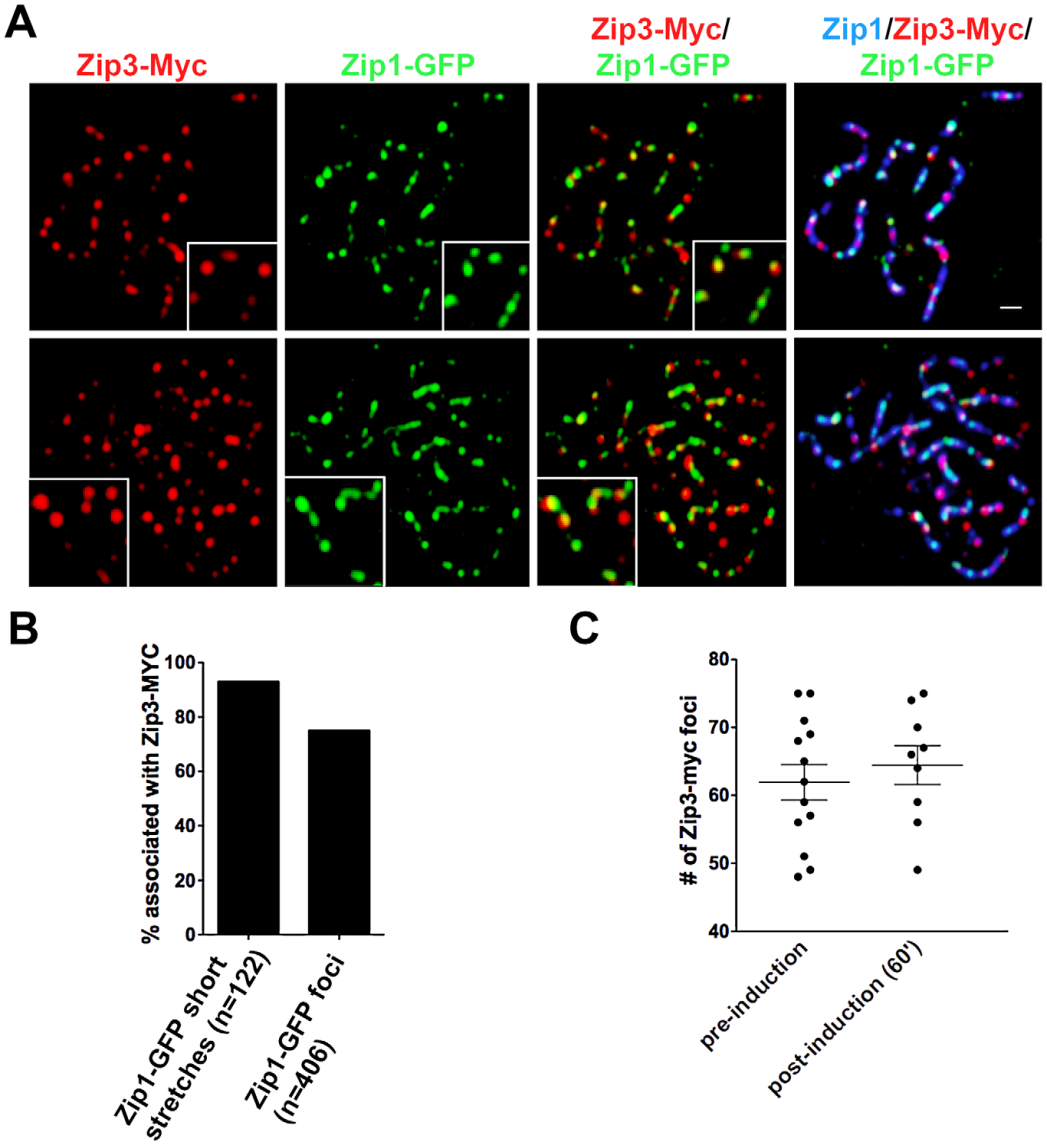
Sites of initial Zip1 entry into full-length SC localize near Zip3 foci. (A) Surface-spread meiotic nuclei from Zip3-MYC strains (K48) after a short induction of *ZIP1-GFP* expression (45 minutes). Zip3-MYC (red) foci often touch or overlap a Zip1-GFP (green) initial deposition event on full-length SCs (anti-Zip1 staining is shown in blue). Insets show 1.5× zoomed regions of the images, to highlight examples of Zip1-GFP localization with Zip3-MYC. Scale, 1 µm. (B) Bar graph indicating the percent of Zip1-GFP short stretches (0.35–0.5 µm) or foci that overlap or localize adjacent to (touching) a Zip3-MYC focus. The scatterplot in (C) displays the number of Zip3-MYC foci exhibited by synapsed chromosomes in nuclei prior to or after *ZIP1-GFP* induction. Circles indicate values for individual nuclei; horizontal lines indicate mean and standard error of the mean.

We first assessed whether sites of initial Zip1-GFP incorporation into full-length SCs co-localize with the Synapsis Initiation Component (SIC), Zip3 [16]. Meiotic nuclei containing fully-synapsed chromosomes and expressing Zip3-MYC were exposed to a short period (approximately 45′) of *ZIP1-GFP* expression before they were harvested for chromosome spreads and immunostained with anti-Zip1, anti-GFP and anti-MYC antibodies. Zip1-GFP and Zip3-MYC distribution was assessed on SCs from nuclei with maximally spread chromosomes. 72% (n = 406) of total Zip1-GFP foci on chromosomes localized directly adjacent to or overlapping (most typical) a Zip3-MYC focus, and 93% (n = 122) of total Zip1-GFP short linear stretches partially overlapped a Zip3-MYC focus (Figure 3). Zip1-GFP induction does not itself cause recruitment of additional Zip3-MYC to chromosomes, as meiotic chromosomes exhibit an equal number of Zip3-MYC foci before and after *ZIP1-GFP* induction (Figure 3C).

In order to assess whether the localization of initial post-synapsis Zip1-GFP incorporation events near Zip3-MYC foci is significant, Monte Carlo sampling analyses with 10^6^ iterations (see Methods) were performed on a subset (n = 69) of well-spread Zip1 stretches that exhibited a range of the smallest GFP foci (0.58 µm or less; average size = 0.31 µm), representing the earliest Zip1-GFP incorporation events (see Figure S6 for examples). Results from Monte Carlo simulations indicated that the observed frequency of post-synapsis Zip1-GFP incorporation events that are completely encompassed by a Zip3-MYC focus (42/117) is significantly higher than expected from a random distribution of Zip1-GFP foci on Zip1 stretches, given the spatial organization of Zip3-MYC foci on each stretch and the dimensions of Zip1-GFP foci randomly taken from the sampled Zip1-GFP population (P = 0.0072). Furthermore, this statistical test indicated that the observed frequency of post-synapsis Zip1-GFP positioned directly adjacent to, partially overlapping with *or* completely encompassed by a Zip3-MYC focus (78/117 for this dataset) is significantly higher than expected from a random distribution (P = 0.008). We conclude that at least a subset of initial post-synapsis Zip1-GFP events preferentially colocalize with Zip3 foci.

**Figure 4:**
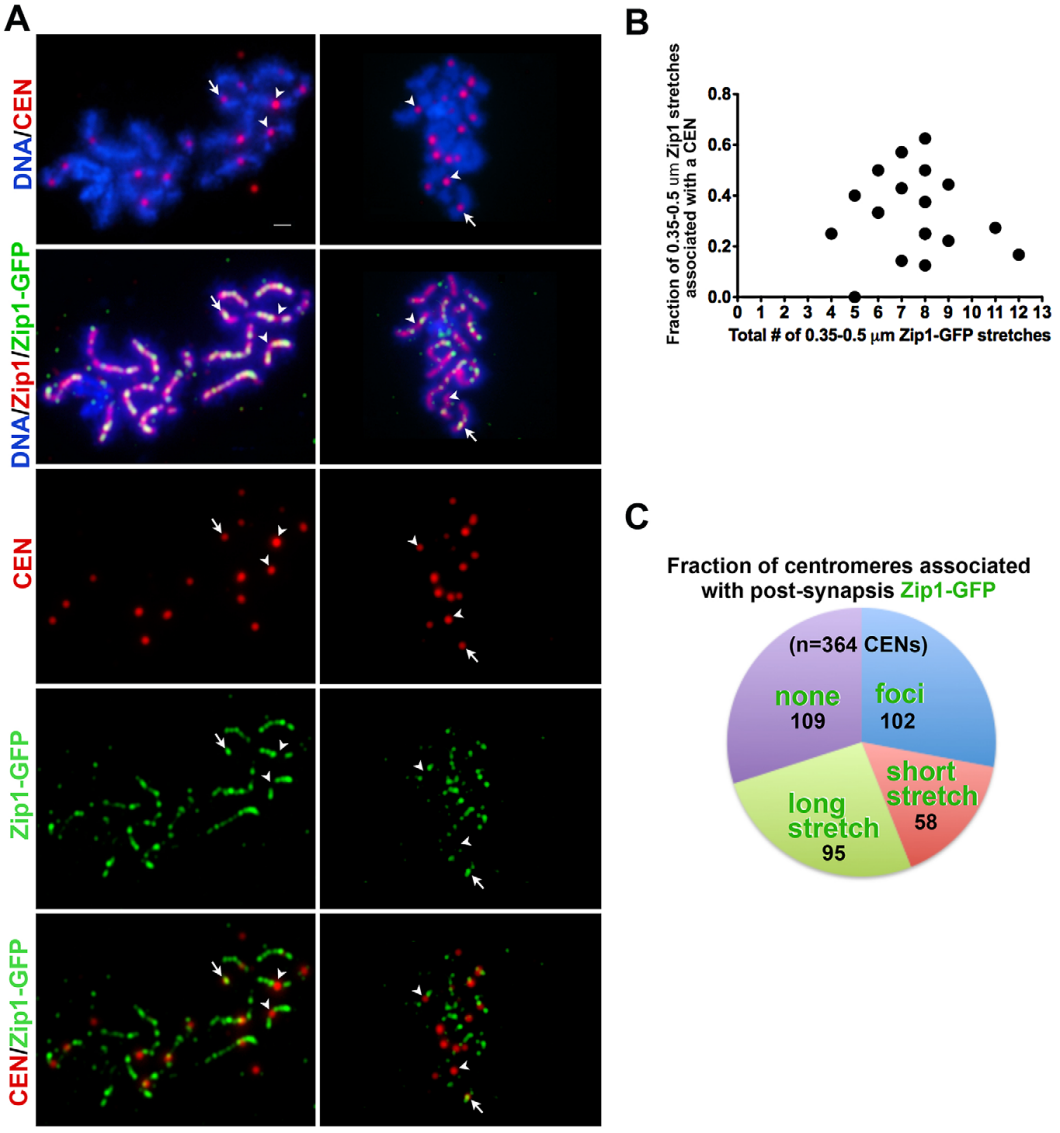
Sites of initial Zip1 entry into full-length SC exhibit no preference for centromere over chromosomal arm sites. (A) Meiotic surface spread chromosomes from K39 strains expressing the centromere marker Ctf19-MYC (red) and Zip1-GFP (green) fixed after 45 minutes of induction. DNA (blue) and anti-Zip1 (red) is shown in the second row. White arrows indicate centromeres with an overlapping Zip1-GFP focus, while arrowheads indicate centromeres devoid of Zip1-GFP. Scale, 1 µm. (B) Zip1-GFP in relation to centromere distribution data from nuclei with earliest Zip1-GFP incorporation events. The X axis of the scatterplot indicates the total number of short Zip1-GFP stretches in each nucleus (circles indicate values for individual nuclei), while the Y axis displays the fraction of these initial incorporation events that are adjacent to or overlapping a centromere. For most nuclei, fewer than half of the Zip1-GFP incorporation events localize with a centromere even when total incorporation events are small. (C) Pie graph displays Zip1-GFP and centromere relative distribution data for nuclei exhibiting robust Zip1-GFP incorporation after 45 minutes of induction. (These nuclei display a number of Zip1-GFP discrete events that outnumber centromeres.) Each color represents the fraction of centromeres in such nuclei associated with Zip1-GFP foci (blue), short stretches (0.35–0.5 µm) (pink), long stretches (>0.5 µm) (green). Centromeres displaying no Zip1-GFP are shown in purple. Actual numbers corresponding to each category are shown on the graph (black).

During wild-type meiotic prophase in the BR1919 strain background, 50–80% of detectable earliest synapsis initiation events occur at centromeres [19]. We assessed whether “post-synapsis” Zip1 incorporation usually occurs first at centromeres by monitoring the distribution of induced Zip1-GFP relative to a tagged centromere protein, Ctf19-MYC (Figure 4). After approximately 45′ of induction, nuclei with earliest Zip1-GFP incorporation events (i.e. nuclei with between 4–12 short GFP stretches) were examined by immunostaining. Among these nuclei, typically fewer than half of short (0.35–0.5 µm) Zip1-GFP entities were localized at or adjacent to a centromere, indicating that centromeres are not completely occupied by Zip1-GFP prior to Zip1-GFP incorporation at chromosome arm sites (Figure 4B). We next examined all centromeres in a population of nuclei exhibiting clear Zip1-GFP induction (>12 discrete Zip1-GFP incorporation events per nucleus). Among centromeres in this population, 28% (n = 364) colocalized with a Zip1-GFP focus, 42% colocalized with a short or long stretch of Zip1-GFP, while 30% of centromeres lacked Zip1-GFP staining (Figure 4C). These data suggest that, centromeres are not necessarily preferred over chromosomal arm sites for initial post-synapsis incorporation of Zip1.

### Sites of Initial Zip1 Incorporation into Previously Established SC Do Not Strictly Reflect Pattern of Zip1 “Peaks” in Full SC

Previous work had suggested a non-uniformity in the structure of the budding yeast SC central region: discrete, local domains of SC central region stain more intensely with Zip1 antibody (Zip1 “peaks”) as compared to other domains (“valleys”) [30]. Using a subset of immuno-stained meiotic nuclei that were maximally surface-spread such that Zip1 staining formed visually-apparent domains of thicker (peaks) and thinner (valleys) Zip1 staining regions along the length of the SC, we explored whether early sites of post-synapsis Zip1-GFP incorporation strictly associate with either “peaks” or “valleys” of a previously deposited SC. We observed a substantial fraction of post-synapsis Zip1-GFP incorporation events (25% (n = 300)) that clearly colocalized with a “valley” of SC central region (Figure S3). The remaining sites either localized at a recognizable “peak” of SC central region (45%) or could not be unambiguously assigned to either an SC “peak” or “valley” (30%). Thus, the distribution of initial Zip1-GFP incorporation sites along the length of the SC does not precisely mimic the global domain structure of Zip1 within the SC itself. This conclusion is further supported by our observation that Zip3-MYC foci localize to Zip1“valleys” as well as to Zip1 “peaks” (Figure S3) and that meiotic cells missing Pch2, a protein that promotes the non-uniform distribution of Zip1 in SC [30], nevertheless exhibit a non-uniform spatial pattern of Zip1-GFP deposition into previously established SCs that looks indistinguishable from *PCH2*+ cells (see below).

### SIC Components, Pph3, and Pch2 Are Dispensable for a Non-Uniform Pattern of Initial Zip1 Incorporation into SC

Our observation that most initial sites of Zip1-GFP incorporation occur at or near Zip3 foci on full-length SCs, in conjunction with previous demonstrations that the SICs, Zip2, Zip3 and Zip4, co-localize on fully synapsed chromosomes, raises the possibility that post-synapsis Zip1 incorporation requires SIC function.

It is technically challenging to address the question of whether SIC function is required for SC maintenance dynamics, since SIC proteins are normally required for SC assembly in the first place. However, while SIC proteins are indispensable for SC assembly at a set of recombination sites along chromosome arms, SC assembly at centromeric synapsis initiation sites is less reliant on SIC function, provided that the Fpr3 and Zip3 proteins are absent [5]. In fact, *zip3* mutant meiotic nuclei assemble a limited amount of SC, using predominantly centromeric synapsis initiation sites, even when Fpr3 is present [16], [19]. Thus we monitored initial Zip1-GFP incorporation into previously established SC in *zip3* single mutants, as well as in meiotic nuclei from *zip3 fpr3, zip2 zip3 fpr3* and *zip4 zip3 fpr3* mutant strains.

**Figure 5:**
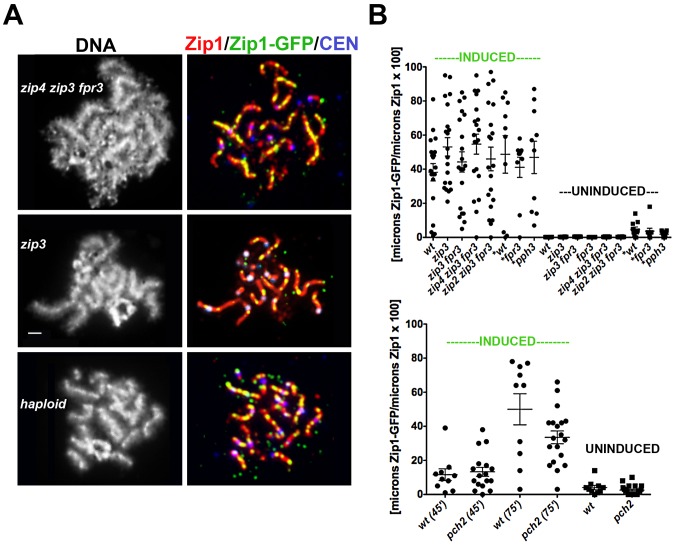
A non-uniform spatial pattern of post-synapsis Zip1 deposition is observed independent of synapsis initiation components, Pch2, Pph3, and the presence of a homolog. (A) Meiotic surface-spread chromosomes labeled with DAPI to show DNA (white, left panels) anti-Zip1 (red), anti-GFP (labels Zip1-GFP, green) and Ctf19-MYC (labels centromeres, bluish purple) to show representative images of Zip1-GFP incorporation (after a 45 minute induction) into previously established SCs in mutants deficient in synapsis initiation components, and in haploid meiosis (K139). Scale, 1 µm. (B) Scatterplot displays the cumulative length of non-focal Zip1-GFP incorporation per µm of Zip1 in nuclei from various mutant strains, under induced (45 minutes) or uninduced conditions. Circles indicate values of Zip1-GFP length per total Zip1 length for individual nuclei; horizontal and error bars indicate mean and standard error of the mean. Complete genotypes of wt (K39), *zip3* (K59), *zip3 fpr3* (K83), *zip4 zip3 fpr3* (K91), *zip2 zip3 fpr3* (K92), *fpr3 (*K84), *pph3 (*AM2560) are listed in Table S1. An analogous graph is depicted below for wild type and *pch2 (*K117) mutant strains; these strains were analyzed independently of the experiments pictured above. All experiments were completed at least twice. (A scatterplot showing the extent of Zip1 assembled in the meiotic nuclei of these various strains at the time of *ZIP1-GFP* induction is shown in Figure S4.).

After a short incubation in β-estradiol (45 minutes), 62%, (n =  1054) of Zip1 stretches exhibited by SIC-defective meiotic nuclei exhibited Zip1-GFP incorporation at multiple discrete sites across their entire lengths, suggesting that new Zip1 incorporation into previously established SCs can occur independent of SIC activity *per se*. Moreover, while SIC-defective mutants exhibited a lower cumulative length of SC as compared to wild type meiotic pachytene nuclei (Figure S4), they exhibited a similar average extent of incorporation of Zip1-GFP per unit length of previously established SC (Figure 5B and Figure S4). While *de novo* synapsis initiation occurs predominantly at centromeric sites in SIC-deficient meiotic cells [5], chromosomal arms exhibit no significant deficit in favored sites of post-synapsis Zip1-GFP incorporation, as seen by the existence of a centromere marker in our immunostained preparations (Figure 5A) and consistent with our measurements above. We note that strains missing Zip2 and Zip4 exhibited a slight reduction in the frequency of discrete Zip1-GFP incorporation events per cumulative length of SC (Figure S4). However the range in the fraction of SC decorated by Zip1-GFP after a 45-minute induction in Zip2-and Zip4-deficient strains (data derived from the same experiments) was similar to wild type (Figure 5B). These observations are consistent with the idea that Zip1-GFP incorporation perhaps occurred slightly faster in Zip2 and Zip4-deficient strains during these experiments, with either a similar or a reduced number of favored sites. Overall, our analyses demonstrate that while post-synapsis Zip1-GFP incorporation initially often localizes adjacent to Zip3, (and by inference, Zip2 and Zip4), SICs are not required, *per se*, for ongoing Zip1 incorporation into previously established SC, nor for the existence of discrete sites of initial Zip1 incorporation.

The Pph3 phosphatase regulates Zip1 phosphorylation status during meiotic prophase [31]. In light of the known role for phosphorylation dynamics in regulating assembly and disassembly dynamics of intermediate filaments [32], [33], we were interested in whether *pph3* mutant cells would exhibit a defect in post-synapsis Zip1-GFP incorporation. The pattern and extent of Zip1-GFP incorporation into full length SCs built in the absence of Pph3 is similar to that displayed by wild-type cells, as shown in Figure 5B. Similarly, SCs built in the absence of the Pch2 protein, which has been proposed to influence the distribution of Zip1 within the SC central region [30] exhibits a non-uniform spatial pattern of Zip1-GFP deposition into previously-established SCs that appears similar to wild-type meiotic nuclei (Figure 5B).

### A Non-Uniform Spatial Pattern of Initial Zip1 Incorporation into Previously Established SC Occurs in the Absence of a Homolog

Since Zip3 are presumed to mark interhomolog crossover recombination intermediates, we asked whether post-synapsis Zip1-GFP incorporation dynamics depend upon an interaction with a homolog by examining Zip1-GFP incorporation into assembled SC during haploid meiosis. *MAT* **a**/*MATα* haploid cells carrying an untagged *ZIP1* gene on a *CEN* plasmid and an inducible *ZIP1-GFP* gene at the chromosomal locus were sporulated for 26 hours and then exposed to β-estradiol to induce *ZIP1-GFP* expression. A fraction of surface-spread nuclei from sporulated haploid cells exhibited extensive Zip1 assembly, albeit with a temporal delay (18/101 nuclei exhibited long Zip1 stretches and 12/101 nuclei displayed Zip1 assembled along the full length of all chromosomes). Although SC assembly in haploid meiotic cells has been reported previously [34], it should be noted that the *ndt80* mutation in our strains may account for the somewhat higher frequency of full-length Zip1/SC stretches observed. After a 45-minute induction, 100% (n = 25) of those haploid meiotic cells containing extensive Zip1 stretches displayed Zip1-GFP deposition at multiple discrete sites along the length of SCs (Figure 5A). Thus, a homolog is dispensable for the establishment of an SC architecture that displays a non-uniform pattern of initial Zip1 entry sites. Interestingly, our studies revealed that Zip1 assembled on haploid chromosomes also exhibits a focal pattern of Zip3 (Figure S5), suggesting the possibility that Zip3-marked recombination structures (presumably involving sister chromatids) exist in haploid meiotic nuclei.

### Zip1 Subunit Loss from Previously Assembled SC

**Figure 6:**
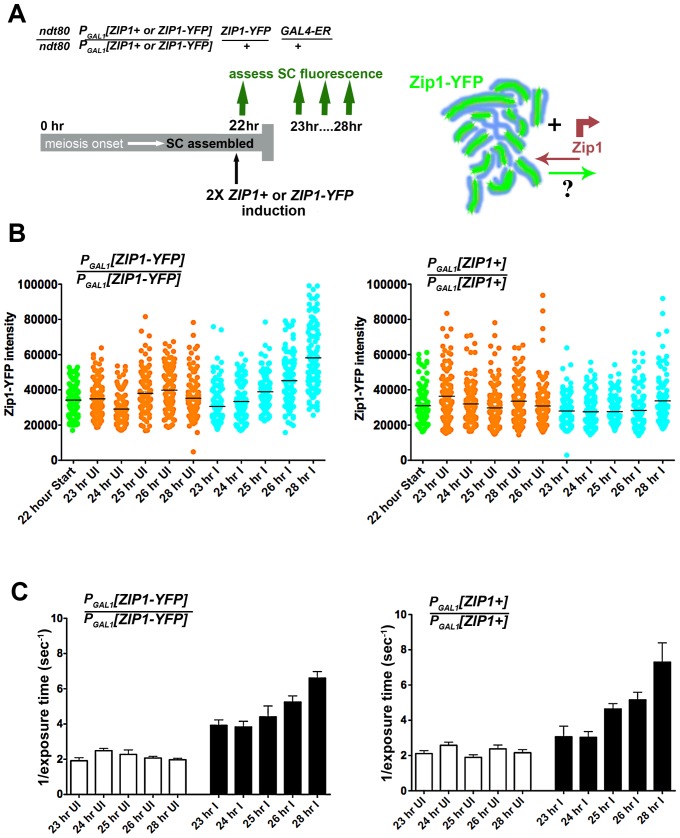
Zip1 subunits do not exit to the same extent that they enter the SC. Cartoon in (A) outlines the induction experiment designed to ask whether Zip1 subunits exit the full-length SC structure. Meiotic nuclei with full length SC built of *ZIP1-YFP* (green) are induced to express two copies of untagged *ZIP1* (SM248) or *ZIP1-YFP* (SM240), at 22 hours of sporulation. SC intrinsic fluorescence is measured at multiple time points after induction. Scatterplots in (B) depict the fluorescence intensity (expressed in arbitrary units) of segments of SCs comprised predominantly of Zip1-YFP. Uninduced datapoints (orange) show a range of SC intrinsic fluorescence in the absence of induced *ZIP1*or *ZIP1-YFP* expression. Induced datapoints (blue) show ranges of SC fluorescence at specific time points after induction of two copies of *ZIP1-YFP* (left) or untagged *ZIP1* (right), starting at 22 hours of sporulation (when most nuclei contain full-length SC). SC intrinsic fluorescence was measured in three adjacent 0.066 µm^3^ ROI volumes, placed over well-spread regions of SCs in multiple nuclei for each time point. Circles indicate values for individual ROI volumes; each time point contains 120 circles. Average fluorescence intensity of Zip1-YFP increased in the strain where *ZIP1-YFP* is induced (blue, left graph), while fluorescence intensity remained similar between time points when untagged *ZIP1* is induced (blue, right graph). While several time points exhibited ranges that were significantly different from the earliest (23 hr) time point (for both uninduced and induced columns), only the 26 hr and 28 hr induced columns from the left graph display a two-tailed P value of <0.0001 (Mann-Whitney test). Horizontal bars indicate the mean. (C) Bar graphs show quantitation of Zip1 levels in SC, measured by intensity of anti-Zip1 antibody, from both strains in uninduced and induced conditions. SC growth in induced strains from both experiments increased to a similar extent despite whether Zip1-YFP (left) or Zip1 (right) was induced. As optimal exposure time of labeled Zip1 is indirectly proportional to the level of Zip1 in SCs, 1 divided by the average exposure time is shown. Optimal exposure times that maximize dynamic range were selected by the *Softworx* application in each case. Error bars indicate the standard error of the mean (n = 10). For (B) and (C), similar results were obtained from 2 independent time course experiments (one experiment is displayed here).

In order to investigate whether Zip1 subunits exit to the same extent as they enter the SC structure, we carried out a complementary version of our first Zip1 induction experiment (Figure 6A). We held *ndt80* mutant cells expressing one chromosomal copy of *ZIP1-YFP* (a kind gift of D. Kaback, constructed as in [35]) at late prophase arrest until a majority of nuclei exhibited full-length, intrinsically fluorescent SC central region (22 hours). Next, we induced the expression of *two* copies of *ZIP1+* using the estrogen-inducible *P_GAL1_* system. In addition, we carried out the same induction experiment using a strain expressing one copy of *ZIP1-YFP* under the endogenous *ZIP1* promoter and additionally carrying two copies of *ZIP1-YFP* under *P_GAL1_* control. Given the fact that Zip1 subunits rapidly incorporate into full-length SCs, we reasoned that if Zip1 exit accompanies entry, we should observe a decrease in the intrinsic fluorescence of Zip1-YFP SCs after some hours of induced Zip1 (untagged) expression. On the other hand, we should observe no such decrease in SC fluorescence in meiotic nuclei from a strain in which *ZIP1-YFP* is induced from two chromosomal loci.

Zip1-YFP fluorescence intensity was sampled in three adjacent 0.25×0.25×1 µm region-of-interest (ROI) volumes (a volume that encompasses the full *z*-dimension of a segment of SC), positioned along the length of a well-spread SC (Figure 6). The range of fluorescence intensities of such ROI volumes did not change significantly for the uninduced strains throughout the time courses. Moreover, cells induced to express two copies of *ZIP1+* exhibited no decrease in the range of SC fluorescence intensities. Interestingly, however, a significant increase (two-tailed P<0.0001; see Figure 6 legend) in the intrinsic fluorescence of SC volumes was observed several hours after induction of expression from two copies of *ZIP1-YFP*. When we quantified the intensity of Zip1 in SCs using an anti-Zip1 antibody that can detect both Zip1 and Zip1-YFP species, both strains exhibited similar, significant increases in the intensity of Zip1 immunofluorescence, presumably representing an increase in SC volume and/or density under induction conditions (Figure 6). Thus, we conclude that Zip1 subunits do not exit the SC structure to the same extent as they enter, and that, as a consequence, the full length SC continuously builds in volume and/or density during a steady state (such as meiotic prophase arrest).

### SC Continues to Build after Its Initial Full-Length Installation

**Figure 7:**
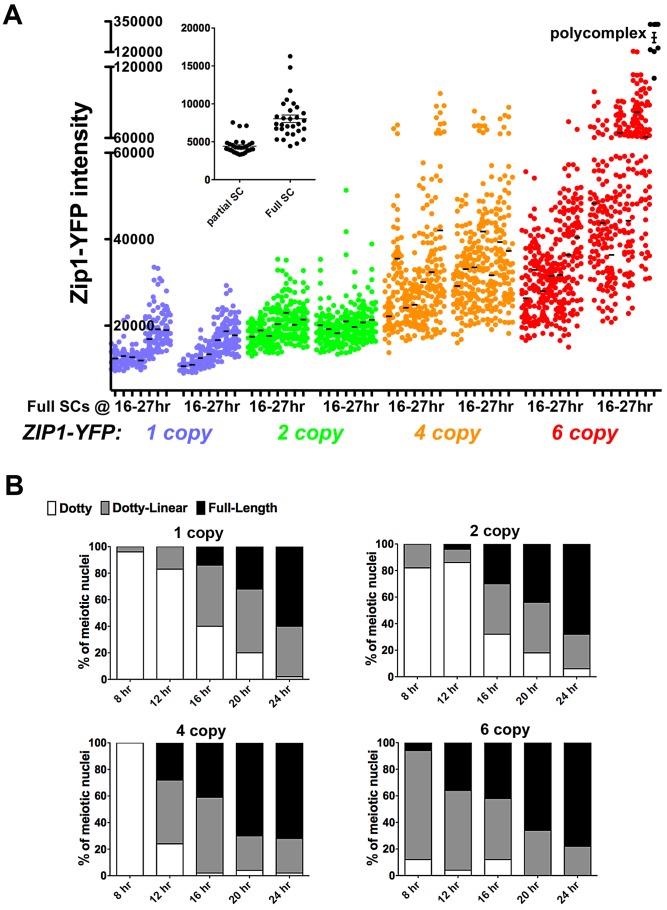
SC builds in a Zip1 concentration-dependent manner. Scatterplot in (A) depicts the fluorescence intensity (in arbitrary units) per 0.066 µm^3^ (0.25×0.25×1 µm) ROI volume encompassing an SC segment. Circles indicate values for individual rectangular volumes, placed across the length of a well-spread SC from a fully synapsed nucleus. Three adjacent rectangular volumes were recorded per SC stretch. The X axis indicates intervals of a time course spanning 16 and 27 hours of sporulation (time points are: 16, 17, 19, 21, 23, 25, 27 hours). Two independent time courses, done via three experiments (see Methods) are plotted for each strain, containing one (blue, SM170), two (green, SM176), four (orange, SM224), or six (red, SM232), copies of *ZIP1-YFP*. Note that the four and six copy strains carry one or two copies of a chromosome III that carries two *ZIP1-YFP* tandem integration events at the *LEU2* locus (determined by southern blot, see Methods). Black circles on far right indicate fluorescence intensities in polycomplex (using the same ROI volume as for SC stretches). Inset graph compares the intensities of the center Z section of an ROI volume stack from full-length versus partial SCs built in *ZIP1-YFP* 2 copy strains. Partial SCs were selected from incompletely synapsed, but well spread nuclei, based on comparing DAPI staining (chromosome territories) with Zip1-YFP distribution. (B) Individual bar graphs depict the extent of SC assembly in strains with a specific *ZIP1-YFP* copy number. For each strain at 8, 12, 16, 20 and 24 hours of sporulation, the extent of SC assembly was characterized as “Dotty” (Zip1 foci only, white), “Dotty Linear” (foci and some linear stretches of Zip1, grey) or “Full-length” (linear Zip1 throughout most all chromosomes, black).

To confirm the observation that steady-state SC grows over time and to explore whether the steady-state SC has an intrinsic size constraint within the context of aligned homologs, we analyzed SC central region size during a time course of meiotic prophase in *ndt80* mutant (thus prophase-arrested) meiotic cells (Figure 7A) from strains carrying *ZIP1-YFP* as their sole source of Zip1. We measured the intensity of Zip1-YFP in ROI volumes of full-length SC (as described above) in cells carrying one, two, four or six copies of *ZIP1-YFP*. All *ZIP1-YFP*-carrying strains exhibited similar sporulation efficiencies (data not shown) and spore viability (Table S2).

Our observations from these experiments support the idea that Zip1 subunits readily enter but rarely exit the budding yeast SC. The range of intensities of Zip1-YFP within a specified ROI volume of full-length SC in all strains increased, on average, during the 11 hours of meiotic prophase sampled during the time course (Figure 7A). Importantly, full-length SCs measured at time points spanning 16–21 hours of sporulation, which likely correspond to periods of “normal” pachytene progression in our BR strain background (as opposed to an “arrested” pachytene state) exhibited progressively larger Zip1-YFP signals per ROI volume. Moreover, at any given time point, full-length SC from strains carrying a larger *ZIP1-YFP* copy number exhibited progressively larger Zip1-YFP signals per ROI volume. This increase in Zip1-YFP signal was evident even between SCs from strains carrying one versus two copies of *ZIP1-YFP*. Thus, even at 16 hours of sporulation, full-length SCs from strains containing two copies of *ZIP1-YFP* typically had experienced some measure of post-synapsis Zip1-YFP incorporation, as these SCs, on average, contain substantially more Zip1-YFP than full-length SCs from strains carrying just one copy of *ZIP1-YFP* (at the same early time point). These data indicate that the budding yeast SC central region exhibits net growth after its initial installation at the interface of homologous chromosomes.

The fact that strains with increased *ZIP1-YFP* copy number displayed correspondingly increased average Zip1-YFP intensity in their full-length SCs at even the earliest time point suggests that meiotic cells carrying more copies of *ZIP1-YFP* generate full-length SCs earlier, on average, than cells with fewer *ZIP1-YFP* copies (and thus have a “head start” on post-synapsis incorporation of Zip1-YFP). Consistent with this idea, we observed a direct correlation between *ZIP1-YFP* copy number and the rate at which chromosomes achieve full synapsis (Figure 7B). (We note that these data do not indicate the mechanism underlying faster achievement of full-length SC in strains carrying additional copies of *ZIP1-YFP*; such a mechanism could involve additional SC initiations or faster elongation from a given initiation site.)

Interestingly, Zip1-YFP polycomplex formation occurred at a frequency of 83% or more in nuclei from the *ZIP1-YFP* 4 and 6 copy strains, even at the earliest time points sampled, yet SC structures still exhibited net growth. This result indicates that the appearance of polycomplex *per se* does not signal a loss of capacity to incorporate into SC.

Within the constraints of our time course experiment we did not observe any upper limit to SC intensity; in fact, during one of the two experiments shown for the strain with 6 copies of *ZIP1-YFP*, select SC regions exhibited intensities approaching that found in Zip1-YFP polycomplex aggregates. Together, these data suggest that a synapsed homolog pair exerts little constraint on SC growth. Furthermore, our untagged Zip1 induction experiment (Figure 6) in conjunction with these growth experiments suggest that SC central region exhibits extremely low subunit turnover; previously deposited SC remains static as additional central region subunits continue to build.

## Discussion

### Zip1 Incorporates into Previously Assembled SC with a Nonuniform Spatial Pattern

Our induction experiments demonstrate that the full-length SC in budding yeast is dynamic, as new transverse filament subunits (Zip1-GFP) continuously incorporate when meiotic cells are arrested at late prophase. It will be informative to learn whether additional proteins that have been implicated in generating SC central region, such as SUMO, exhibit similar post-synapsis incorporation dynamics.

Instead of a uniform incorporation of Zip1-GFP along the length of a previously established SC, post-synapsis Zip1-GFP subunit incorporation initially exhibits a focal pattern. Such initial Zip1 entry sites may occur at centromeres and a majority of them localize adjacent to sites marked by the synapsis initiation component, Zip3. Our statistical analysis indicates that Zip3 marks at least a subset of initial post-synapsis Zip1-GFP incorporation sites.

Our observation that at least a subset of post-synapsis Zip1-GFP incorporation events preferentially localize to Zip3-marked sites along the SC raises the possibility that many or all of the ongoing Zip1-GFP incorporation events into full-length SC could be the result of synapsis initiation activity by SIC proteins, as the *de novo* initiation of Zip1 assembly (synapsis) at presumed recombination sites during normal meiotic progression requires the SIC proteins Zip2, Zip3 and Zip4. On the contrary, post-synapsis Zip1 incorporation sites do not exhibit the same regulatory mechanisms as sites of *de novo* synapsis initiation, since our data demonstrate that post-synaptic events are not dependent on Zip2, Zip3 or Zip4 proteins (Figure 5, Figure S4).

An interesting possibility is that favored Zip1 entry sites contribute to the establishment of Zip1 “peaks” and “valleys” in full length SC. However, under such a model one might expect that post-synapsis Zip1-GFP incorporation events would seldom localize to Zip1 “valleys”. We observed that sites of post-synapsis Zip1 incorporation do not strictly correspond to either local peaks or valleys of Zip1 within the previously deposited, full-length SC, and the Pch2 protein which regulates such domains of high and low Zip1 [30] does not alter the frequency or qualitative pattern of Zip1 incorporation sites. One could reconcile our observation that a fraction of initial post-synapsis Zip1-GFP events occur at Zip1 valleys with the idea that favored Zip1 incorporation sites contribute to the formation of Zip1 peaks if we further propose that rates of Zip1-GFP incorporation varies between favored sites or at a given site over time. Alternatively, Zip1 peaks and valleys may arise from a mechanism that mediates Zip1 subunit movement laterally within the SC.

Favored Zip1 entry sites are intriguing because they indicate a level of non-uniformity in SC architecture. However, with time, the pattern of post-synapsis Zip1-GFP distribution completely overlaps the full-length SC. One explanation for the progressive change in the distribution of post-synapsis Zip1 might be that the budding yeast SC maintains a capacity to add new Zip1 all along its length but certain sites are more favorable for addition than others. Alternatively, as mentioned above, perhaps the SC incorporates new transverse filament subunits exclusively at certain sites but that some newly incorporated subunits can move to a different location along the length of the SC. Indeed, the frequent occurrence of post-synapsis Zip1-GFP foci that only partially overlap Zip3-MYC gives the impression that newly incorporated subunits might grow outward from the Zip3-MYC domain. Such immunofluorescence data, however, are also consistent with a model in which post-synapsis Zip1-GFP domains grow longer via continued incorporation at both Zip3 and (eventually) non-Zip3 sites.

### Transverse Filaments Exhibit Robust Entry into but Minimal Exit from Full-Length SC during Pachytene Arrest

Our data indicate that, once the SC has assembled along the full length of meiotic axes in cells experiencing pachytene arrest, Zip1 subunits exhibit little turnover but continue to incorporate, allowing the SC to grow to many times its original density or volume. Thus, the SC central region appears to behave more like a “tether” than a “treadmill”, at least during late prophase when chromosomes are fully synapsed.

The absence of significant turnover of previously deposited transverse filaments suggests that the SC may only have the capacity to remodel through new subunit addition. One can imagine that continuous SC assembly might facilitate at least some mechanistic aspects of synaptic adjustment, where an unsynapsed chromosomal domain, contiguous with an otherwise synapsed chromosome pair, eventually incorporates into the full length SC during prophase [2], [36]. However, continuous assembly without SC subunit turnover raises the question of whether budding yeast can correct inappropriate synapsis. Perhaps SC assembly in budding yeast is sufficiently regulated at the outset so that irrevocable synapsis mistakes occur at an extremely low frequency. On the other hand, an SC disassembly mechanism that does not rely on ongoing central region turnover may exist that can be deployed over a local chromatin domain. Another possibility may be that SC central region can be physically disrupted, given enough applied force, without an active disassembly mechanism. Finally, the balance between subunit entry and exit may differ at different stages of budding yeast synapsis, for example during zygotene and early pachytene stages, potentially allowing error correction at those stages before solidifying a final synapsis configuration.

Couteau and Zetka observed local SC central region disassembly in response to irradiation in *C. elegans* late meiotic prophase nuclei [37], suggesting that *C. elegans* SC may have the capacity to remodel in response to recombination intermediates. Contrary to this picture, a relatively static central region in conjunction with ongoing Zip1 incorporation preferentially at Zip3 sites raises the possibility that the budding yeast SC only structurally accommodates those (or a subset of those) recombination events arising prior to or concomitant with SC deposition. This possibility is consistent with several recent models for meiotic prophase chromosome dynamics in budding yeast [38], [39]. Under this scenario, the budding yeast SC central region may not need to “remodel” to structurally accommodate recombination sites, since those that will interface with the SC will have already progressed to a certain intermediate stage prior to SC installation. Such an intermediate stage would need to involve establishing a local environment in which recombination-associated structural changes in the DNA and recombination enzyme complexes can proceed unperturbed by ongoing deposition of SC central region proteins in the surrounding vicinity. We note, however, that our observation of little global SC turnover does not rule out the possibility that SC turnover occurs differentially at distinct sites. As SC demonstrates a non-uniformity in assembly dynamics in the vicinity of recombination events, SC subunit turnover may be higher at preferred entry sites but still below the detection level of our assay.

### Recombination and the Dynamic Architecture of the Budding Yeast SC

The pattern of initial post-synapsis Zip1 incorporation near Zip3 foci, together with the fact that SC assembly is contingent upon early steps in the recombination pathway, suggests that recombination-based events establish much of the non-uniform architecture of the SC. One way to think about how the recombination landscape may shape an SC composite architecture is that certain recombination events create a meiotic axis perturbation that similarly interrupts the SC, and at these interruptions SC dynamics are distinct from those in the rest of the structure.

Importantly, any model attempting to explain the non-uniform dynamics of SC architecture in terms of recombination events must account for the fact that interhomolog crossover recombination intermediates are not required for establishing such SC architecture. Haploid nuclei that have been genetically “tricked” into entering meiosis can build extensive SC (albeit with a temporal delay), and we observe that initial Zip1-GFP incorporation into the previously assembled SCs in haploid meiotic cells occurs with similar timing and with a qualitatively similar distribution as compared to post-synapsis Zip1-GFP incorporation into diploid SCs. We suggest that initial sites of post-synapsis Zip1 incorporation into haploid SCs reflect a set of recombination intermediates analogous to those that we propose shape SC dynamics in diploids, but which engage the sister chromatid. Interestingly, Zip3-GFP or Zip3-MYC decorates the length of SCs in haploid nuclei (Figure S5), consistent with the idea that an analogous set of SIC-associated recombination events, albeit between sister chromatids, influences the architecture of Zip1 structures that are assembled on haploid meiotic chromosomes.

The assembled SC is the context within which at least a subset of meiotic recombination events mature. Moreover, the SC influences the resolution of recombination events. Budding yeast SC-deficient mutants initiate recombination, but the fraction of those double-strand breaks that are repaired to a crossover outcome is diminished [17], [18], [23], [24], and the remaining interhomolog crossovers exhibited by such mutants do not exhibit interference [16]–[18], [24], [38], [40]. Interference refers to a nonrandom distribution such that two crossover events rarely occur close together. Interestingly, Fung et al. demonstrated that chromosome axes display a cytological manifestation of interference, in the form of a nonrandom distribution of synapsis initiation complexes, even in the absence of assembled SC [41]. Yet genetic studies on SIC-deficient meiotic mutants suggest that assembled SC is required to generate interfering interhomolog crossovers. These conflicting observations are reconciled by proposing that assembled SC influences the repair outcome of a set of interfering recombination intermediates. As Zip3 sites have been proposed to mark such a set of interfering recombination intermediates, favored sites of post-synapsis Zip1 incorporation into assembled SC (which largely appear near Zip3) may reflect functional interfaces between assembled SC and crossover-designated recombination intermediates that play a role in maintaining the designation of interfering crossover-destined recombination intermediates and/or play a role in influencing the repair outcome of the associated recombination events.

Finally, ongoing SC growth may itself be functionally important for creating a rigid structure that aids in maintaining or influencing the repair outcome of interfering, crossover-destined recombination intermediates. Under this model, our observation that full-length SCs grow continuously in a Zip1 concentration-dependent manner leads to the prediction that meiotic cells carrying a larger Zip1 copy number will maintain an increased capacity to impose interference. Klutstein et al. (2009) recently reported evidence in support of this prediction; these authors discovered that crossover interference is reduced in strains carrying just a single copy of *ZIP1* [42].

## Methods

### Strains

All diploids are isogenic with BR1919-8B [43]. Strains used in this study are listed in Table S1. Yeast genetic manipulations were carried out via standard procedures. Meiosis-competent haploid strains were constructed using pB211 to integrate *MAT* **a** at *THR1* in a *MATα* haploid [44]. The *ZIP1-GFP* fusion construct used for all experiments except for those measuring fluorescence intensity (Figure 6 and Figure 7) is described in [25]. Fluorescence intensity experiments used *ZIP1-YFP* fusions, in which YFP is inserted between amino acids 700 and 701 of Zip1, as described in [35]. Both fusion constructs were a kind gift of David Kaback. To make strains carrying multiple copies of *ZIP1-YFP*, a *ZIP1-YFP* fragment was excised from *pRS316-ZIP1-YFP* and placed into the *LEU2*-marked integrating shuttle vector pRS305 [45]. The verified clone, BAM179, was cut with Xcm1 in order to target to *leu2*. Integration and function of *ZIP1-YFP::LEU2* was verified by sporulation rescue of a *zip1* null and visual assay of fluorescent SCs in live cells.

### Induction Experiments

The *TRP1::P_GAL1_* promoter cassette was placed upstream of the *ZIP1* or *ZIP1-GFP* ORF by directed transformation of a PCR product with homology to the 5′ end of *ZIP1*. pKB80 (*GAL4.ER::URA3*) was integrated at *ura3* to introduce the chimeric protein that responds to β-estradiol and activates *P_GAL1_* promoters [26]. Strains were grown overnight in YPADU media to late log/early stationary phase at 30°, washed with an equal volume of water and suspended at a 4-fold dilution in 2% potassium acetate (pH 6–6.5). Strains were induced with 1 µm β-estradiol (Sigma E2257, prepared in ethanol). Uninduced cultures were removed from the sporulating culture just prior to induction, and to these cultures an appropriate volume of 95% ethanol was added. 5–10 ml of sporulating culture was removed at various time points for chromosome spreads or TCA protein preparation.

### Cytological Analysis and Imaging

Meiotic chromosome spreads, staining and imaging were carried out as previously described [46] with the following modifications: 80 µl 1xMES and 200 µl 4% paraformaldehyde fix were added to spheroplasted, washed cells, then 80 µl of resuspended cell solution was put directly onto a frosted slide and cells were distributed over the entire slide using the edge of a coverslip with moderate pressure. The slide was allowed to air dry until less than half of the liquid remained, and then washed in 0.4% Photo-flo as described [46]. The following primary antibodies were used: chicken anti-GFP (1∶100) (Abcam), mouse anti c-myc (1∶200) (Invitrogen, 9E10.3), affinity purified rabbit anti-Zip1 (1∶100) (raised at YenZym Antibodies, LLC, against a C terminal fragment of Zip1 as described in [10]). Secondary antibodies were obtained from Jackson ImmunoResearch and used at a 1∶200 dilution.

The time course experiments in Figure 7 were carried out over a series of three experiments each containing at least two strains: experiment 1 (which is depicted first of the two datasets for each strain along the X axis in Figure 7) contained all four strains (1 copy, 2 copy, 4 copy and 6 copy); experiment 2 contained the second dataset for the Zip1 1 copy and the Zip1 6 copy strain; experiment 3 contained the second dataset for the Zip1 2 copy and the Zip1 4 copy strain.

Imaging was carried out using a Deltavision RT imaging system (Applied Precision) adapted to an Olympus (IX71) microscope. Zip1, Zip1-GFP and Zip3-MYC lengths were measured using the Softworx Measure Distance Tool. Graphpad Prism software was used for scatterplot generation and statistical analysis.

All slides used for quantitative fluorescence analysis were assayed for intensity of the native fluorophore (Zip1-YFP) and were stained only with DAPI to visualize DNA. Samples prepared for fluorescence quantitation experiments were imaged with a fixed exposure time so that intensity measurements could be compared between time points and strains. For experiments not requiring fluorescence quantitation, exposure times were optimized on a slide-by-slide basis to obtain linear range.

All fluorescence quantitation experiments used the following imaging conditions. 7 Z-stack sections of 0.2 µm were collected in the FITC channel using a 1 second exposure per section. This exposure time was selected based on the minimal time to achieve a quantifiable fluorescent image, above background levels, in the dimmest SCs (early synapsis in a strain carrying only one copy of *ZIP1-YFP*). As reference, an additional DAPI channel image was acquired at the middle section, with a 0.5 second exposure. Projections were constructed from raw Z-stack data by building a summed-intensity projection of the 5 best-resolved 0.2 µm sections of the Z-stack (usually the 5 middle sections). The intensity in the FITC channel along well-spread SCs was analyzed by using the *Softworx* Data Inspector tool. Three adjacent 0.2572×0.2572 µm square region-of-interest boxes were placed over the region of the SC to be analyzed in the summed-intensity projection. The total intensity of the region of interest (ROI), in units of arbitrary fluorescence intensity, was recorded. The ROI for all intrinsic fluorescence measurements were three-dimensional volumes of 0.066 µm^3^, calculated as follows: 1 pixel = 0.0643 µm; 4 pixels = 0.2527 µm (length of side of 4×4 pixel box); Area of box = (0.2572 µm)^2^ = 0.06615 µm^2^; Volume of a projected box (5 sections of 0.2 µm) = (0.06615 µm^2^)(5 sections)(0.2 µm/section) = 0.066 µm^3^).

### Statistical Analysis

Monte Carlo simulations were done to assess the statistical significance of the distribution of Zip1-GFP foci, relative to Zip3-MYC foci, on 69 well-spread Zip1 linear stretches exhibiting Zip1-GFP foci whose largest dimension spanned 0.58 µm or less of the Zip1 stretch (average size = 0.31 µm). Two distinct analyses were performed. First, we analyzed “adjacency”, defined as the presence of a Zip1-GFP focus directly adjacent to (touching), partially overlapping, or completely encompassed by a Zip3 focus. In this case, the SC domain in which a Zip1-GFP focus would be considered adjacent to, partially overlapping with or encompassed by a Zip3 focus corresponds to the length of the Zip3-MYC focus plus half of the length of a Zip1-GFP focus on each side of the Zip3 focus. For the second analysis, we analyzed “complete overlap”, defined as Zip1-GFP completely encompassed by a Zip3-MYC focus. In this case, the SC domain in which a Zip1-GFP focus would be considered completely overlapping corresponds to the length of the Zip3-MYC focus minus half the length of a Zip1-GFP from the boundaries of that Zip3 focus. Both analyses followed a similar procedure. First, the size of Zip1-GFP is randomly sampled from the experimental data (117 distinct foci). For each of 69 Zip1 stretches of any given size, Zip1-GFP foci of that fixed size are randomly distributed, considering the observed coordinates of Zip3-MYC foci and limiting the allowed number of Zip1-GFP foci to the observed number of Zip1-GFP foci on a particular stretch (i.e. if a given Zip1 stretch displayed three Zip1-GFP foci, each random distribution procedure will place three Zip1-GFP foci onto that stretch). For a generated random distribution, the total number of Zip1-GFP foci falling within the SC domain of “adjacency” or “complete overlap” on the 69 Zip1 stretches is calculated, out of a possible 117. This random assignment of Zip1-GFP foci of the same fixed size on the 69 Zip1 stretches, constraining the allowed number of Zip1-GFP foci on a given Zip1 stretch to the observed number of Zip1-GFP foci on that stretch, is performed 1000 times. Next, a new Zip1-GFP size is randomly selected from the observed Zip1-GFP focus sizes, and the aforementioned steps are repeated. In total, 1000 random samplings of Zip1-GFP sizes are performed, bringing the total number of individual iterations to 1,000,000. The observed number of adjacent Zip1-GFP foci (78/117) or completely encompassed (42/117) is compared to the number of iterations in which they were equal or greater, thus the reported p-value represents the fraction of random iterations in which the number of adjacent/encompassed Zip1-GFP was equal or superior to the observed frequency.

### Western Blot

Protein pellets were isolated by TCA precipitation using 10 mL of sporulating cell culture [15]. The final protein pellet was resuspended at a concentration of 10 µg/µl in 2× Laemmli sample buffer supplemented with 30 mM DTT. Protein samples were heated for 10 minutes at 65°, centrifuged at top speed and 150 µg was loaded onto an 8% polyacrylamide/SDS gel. Proteins were transferred to Whatman Protran nitrocellulose membrane. Rabbit anti-Zip1 antibody was used at 1∶2500 dilution and Alkaline Phosphatase-conjugated AffiniPure Donkey anti-Rabbit (Jackson ImmunoReasearch) was used at 1∶2500 dilution.

### Southern Blot

Yeast genomic DNA was digested with BglII (NEB) overnight at 37°C and separated on a 0.8% agarose gel. Subsequent transfer to nitrocellulose membrane (Roche) was carried out by the alkali method described in Sambrook and Russell [47]. A 1.8 Kb PCR fragment containing the *LEU2* gene was used as a probe against genomic DNA, using DIG High Prime DNA Labeling and Detection Starter Kit II (Roche). Membrane-bound probe was visualized by incubating with an alkaline phosphatase-conjugated anti-DIG antibody followed by BCIP/NBT addition in alkaline phosphatase buffer. Genomic DNA without an integrated *ZIP1-YFP* plasmid gives a 2.8 Kb band whereas one, two, or three *ZIP1-YFP* integration events are predicted to result in a 12.7 Kb, 22.6 Kb, or 32.5 Kb band, respectively.

## Supporting Information

Figure S1 An inducible Zip1 system. Table in (A) gives sporulation efficiency and spore viability of wild type (1919) and homozygous *P_GAL1_[ZIP1] NDT80+ GAL4.ER* (K62) strains (in which *ZIP1* expression is driven by an inducible promoter at its endogenous locus), with or without extended incubation with β-estradiol. Images in (B) show three examples of surface-spread chromosomes from homozygous *P_GAL1_[ZIP1]* meiotic nuclei sporulated in the absence of β-estradiol, immunostained for the meiotic axis protein, Red1 (red) and for Zip1 (green). In these uninduced nuclei, Zip1 signal is rare, and axial associations (arrows) are visible owing to the absence of Zip1 at the interface between aligned homologous axes. The nucleus at far right is one taken from meiotic cultures sporulated in the presence of β-estradiol, thus clear assemblies of Zip1 are visible at the interface between aligned chromosome axes. Scale, 1 µm.
(PDF)

Figure S2 Late prophase meiotic axes are competent to assemble Zip1. (A) Cartoon depicts the “late Zip1 induction” experiment conducted. In this experiment *ZIP1* expression is prevented until either 20 or 26 hours of sporulation. Homozygous *P_GAL1_[ZIP1+] ndt80 GAL4.ER* (K40), cells were sporulated in the absence of β-estradiol for 20 or 26 hours, time points during meiotic prophase arrest where the majority of *ZIP1+* cells would have completed synapsis. β-estradiol was added to sporulating cultures to induce *ZIP1* expression, and nuclei were surface-spread and analyzed at four successive hourly intervals following β-estradiol addition. (B) Bar graphs show that for either experiment, a substantial percentage of nuclei (n>50 for each time point) exhibited short and long Zip1 stretches assembled at the interface of aligned chromosome axes; moreover some nuclei appeared to have completed synapsis (“full SC”; red). Meiotic surface-spread chromosomes from such strains are shown in (C) labeled with anti-Red1 (red) and anti-Zip1 (green) antibodies. Scale, 1 µm.
(PDF)

Figure S3 Zip1-GFP incorporates into full-length SC both at sites of high and sites of low Zip1 abundance. Graph in (A) gives the number of discrete Zip1-GFP incorporation events (focus or short (0.35–0.5 µm) stretch) or Zip3-MYC foci that co-localized with an area of high Zip1 abundance (“Zip1 Peak”), low Zip1 abundance (“Zip1 Valley”), or at the terminus of a synapsed chromosome (“Zip1 Termini”) in K48. Zip1-GFP incorporation events or Zip3-MYC foci that could not be unambiguously assigned to a particular feature of the SC were grouped in the “Ambiguous” category. Images in (B) show examples of Zip1-GFP events (green, top two rows) or Zip3-MYC foci (green, bottom row) in Zip1 (red) valleys (arrows) or peaks (arrowheads). DNA for these nuclei is shown in white (at left). Scale, 1 µm.
(PDF)

Figure S4 Zip1-GFP incorporation into full length SC may be reduced in situations where the crossover: Zip1 length ratio is predicted to be low. Scatterplots in (A) show the extent of Zip1 SC present in nuclei, upon *ZIP1-GFP* induction (at “start”), for the various strains involved in the incorporation experiment shown in Figure 5. Scatterplot in (B) depicts the number of discrete Zip1-GFP incorporation events (either a focus or a Zip1-GFP stretch) per cumulative length of Zip1, after a 45-minute induction of *ZIP1-GFP* expression. Circles indicate Zip1-GFP incorporation events per cumulative length of Zip1 for individual nuclei. Horizontal and error bars indicate mean and standard error of the mean for each column. The average number of Zip1-GFP incorporation events per µm of Zip1 is significantly reduced (compared to wild type, Mann-Whitney test) for the following strains: *zip2 zip3 fpr3* (two-tailed P = 0.0005) and *zip4 zip3 fpr3* (two-tailed P = 0.0032). The *fpr3* strain also exhibited a significant difference from wild type (two-tailed P = 0.0113), likely due to the longer cumulative lengths of Zip1 exhibited by this single mutant (see A).
(PDF)

Figure S5 Zip3 foci decorate Zip1 stretches in haploid meiotic cells. Meiotic chromosomes (white) from haploid *MAT*a/*MATα* cells carrying Zip3-GFP (AM2632, top row) or Zip3-MYC (K150, bottom row) were sporulated for 26 hours, and then surface spread on glass slides. Zip1 (red) and Zip3-GFP or Zip3-MYC (green) are depicted in single channels and as a merged image. Scale, 1 µm.
(PDF)

Figure S6 Sites of initial Zip1 entry into full-length SC localize near Zip3 foci. (A) Three surface-spread meiotic nuclei (DNA for each nucleus is shown in white, top row) from Zip3-MYC strains (K48) after a short induction of *ZIP1-GFP* expression (45 minutes). Zip1 staining (second row, blue) shows full length SC decorated by Zip3-MYC (red) foci. Bottom row displays several zoomed Zip1 SC stretches (blue) from each nucleus (4–5 per nucleus), which are a subset of the 69 stretches used in our statistical analysis of post-synapsis Zip1-GFP distribution relative to Zip3-MYC. Both Zip3-MYC (red) and post-synapsis Zip1-GFP incorporation events (green) are shown in zoomed images. Scale, 1 µm.
(PDF)

Table S1 Strains used in this study.
(PDF)

Table S2 Viability of spores produced by diploids carrying one to six copies of *ZIP1*. The far right column shows the overall spore viability for each strain. Displayed in each “Distribution of tetrad types” column is the frequency of tetrads containing four viable spores (4-sv), three viable spores (3-sv), two viable spores (2-sv), one viable spore (1-sv) or no viable spores (0-sv). Strain genotypes are listed in Table S1.
(PDF)
