# Supplementary material for: Full-Length Synaptonemal Complex Grows Continuously during Meiotic Prophase in Budding Yeast
Source: PLoS Genet. 2012 Oct 11;8(10):e1002993. doi: 10.1371/journal.pgen.1002993 (PMC3469433; doi:10.1371/journal.pgen.1002993)

Figure S1

A

Genotype	β-estradiol (duration)	4-spore ascii (n)	viable spores (n)
WT	-	57% (123)	99% (88)
WT	+ (91 hr)	49% (131)	93% (88)
$\frac{P_{GAL1}[ZIP1]}{P_{GAL1}[ZIP1]}\ \frac{GAL4\text{-}ER}{+}$	-	7% (127)	59% (88)
$\frac{P_{GAL1}[ZIP1]}{P_{GAL1}[ZIP1]}\ \frac{GAL4\text{-}ER}{+}$	+ (91 hr)	55% (177)	86% (88)
$\frac{P_{GAL1}[ZIP1]}{P_{GAL1}[ZIP1]}\ \frac{GAL4\text{-}ER}{+}$	+ (6 hr)	55% (141)	98% (104)

B

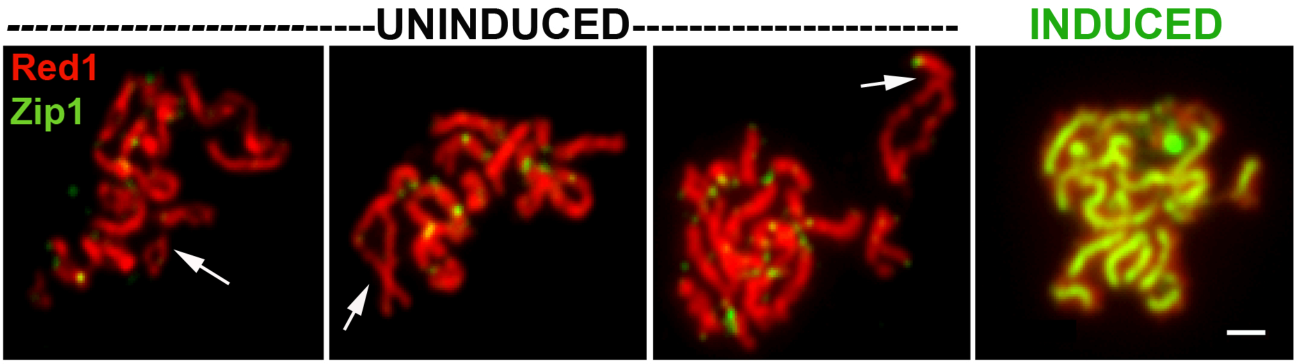

Supplement: Figure S1 — An inducible Zip1 system. Table in (A) gives sporulation efficiency and spore viability of wild type (1919) and homozygous PGAL1[ZIP1] NDT80+ GAL4.ER (K62) strains (in which ZIP1 expression is driven by an inducible promoter at its endogenous locus), with or without extended incubation with β-estradiol. Images in (B) show three examples of surface-spread chromosomes from homozygous PGAL1[ZIP1] meiotic nuclei sporulated in the absence of β-estradiol, immunostained for the meiotic axis protein, Red1 (red) and for Zip1 (green). In these uninduced nuclei, Zip1 signal is rare, and axial associations (arrows) are visible owing to the absence of Zip1 at the interface between aligned homologous axes. The nucleus at far right is one taken from meiotic cultures sporulated in the presence of β-estradiol, thus clear assemblies of Zip1 are visible at the interface between aligned chromosome axes. Scale, 1 µm. (PDF) [file pgen.1002993.s001.pdf]
