# Supplementary figures and images for: Full-Length Synaptonemal Complex Grows Continuously during Meiotic Prophase in Budding Yeast
Source: PLoS Genet. 2012 Oct 11;8(10):e1002993. doi: 10.1371/journal.pgen.1002993 (PMC3469433; doi:10.1371/journal.pgen.1002993)

Figure S2

**A**

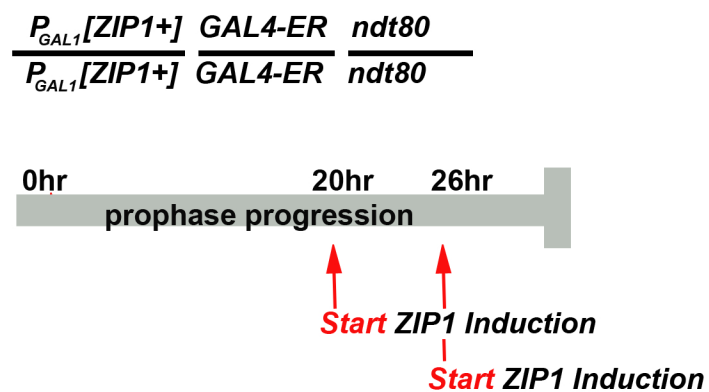

**B**

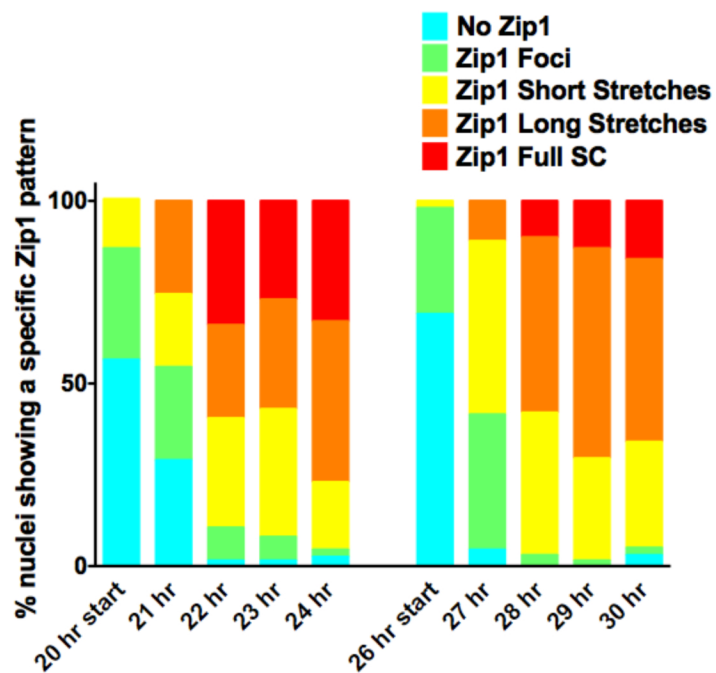

**C**

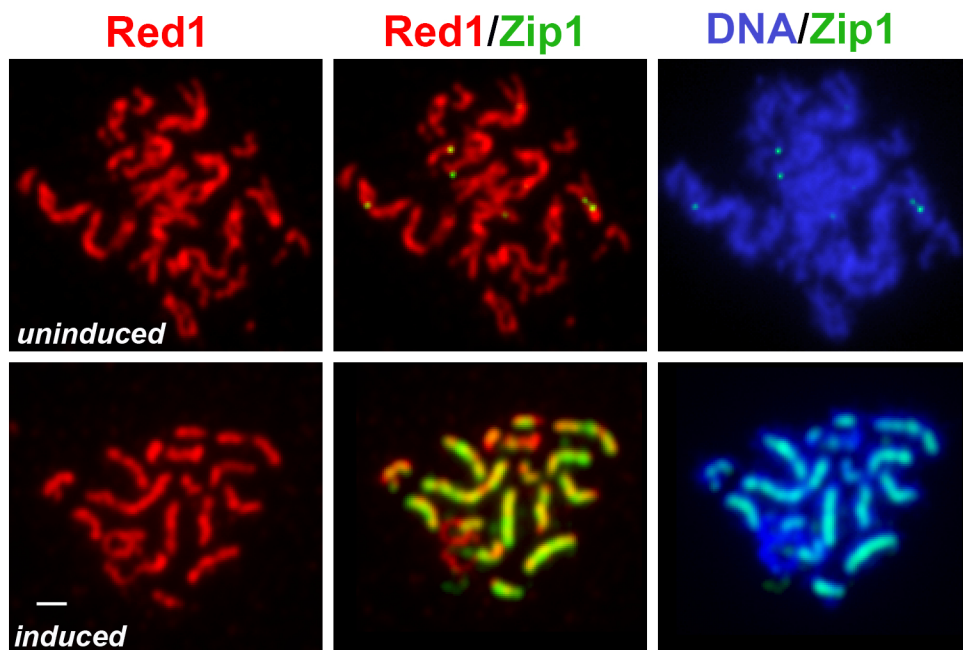

Supplement: Figure S2 — Late prophase meiotic axes are competent to assemble Zip1. (A) Cartoon depicts the “late Zip1 induction” experiment conducted. In this experiment ZIP1 expression is prevented until either 20 or 26 hours of sporulation. Homozygous PGAL1[ZIP1+] ndt80 GAL4.ER (K40), cells were sporulated in the absence of β-estradiol for 20 or 26 hours, time points during meiotic prophase arrest where the majority of ZIP1+ cells would have completed synapsis. β-estradiol was added to sporulating cultures to induce ZIP1 expression, and nuclei were surface-spread and analyzed at four successive hourly intervals following β-estradiol addition. (B) Bar graphs show that for either experiment, a substantial percentage of nuclei (n>50 for each time point) exhibited short and long Zip1 stretches assembled at the interface of aligned chromosome axes; moreover some nuclei appeared to have completed synapsis (“full SC”; red). Meiotic surface-spread chromosomes from such strains are shown in (C) labeled with anti-Red1 (red) and anti-Zip1 (green) antibodies. Scale, 1 µm. (PDF) [file pgen.1002993.s002.pdf]

Figure S3

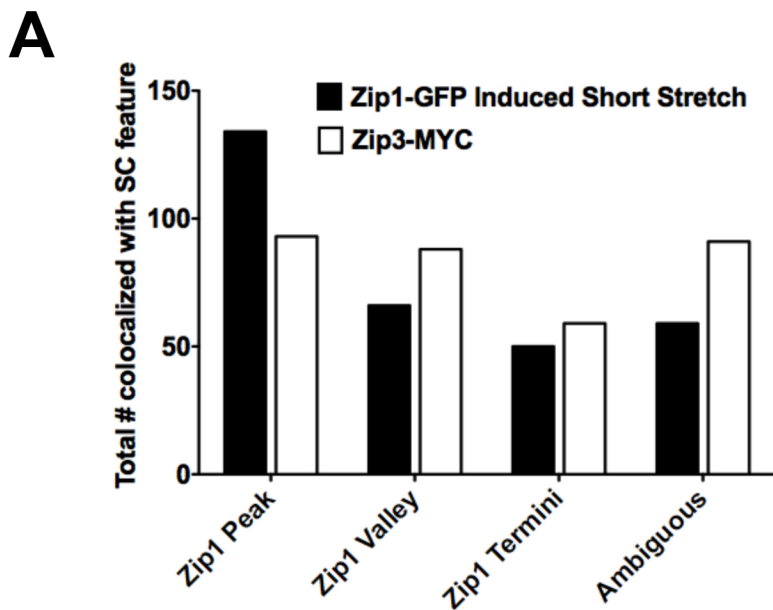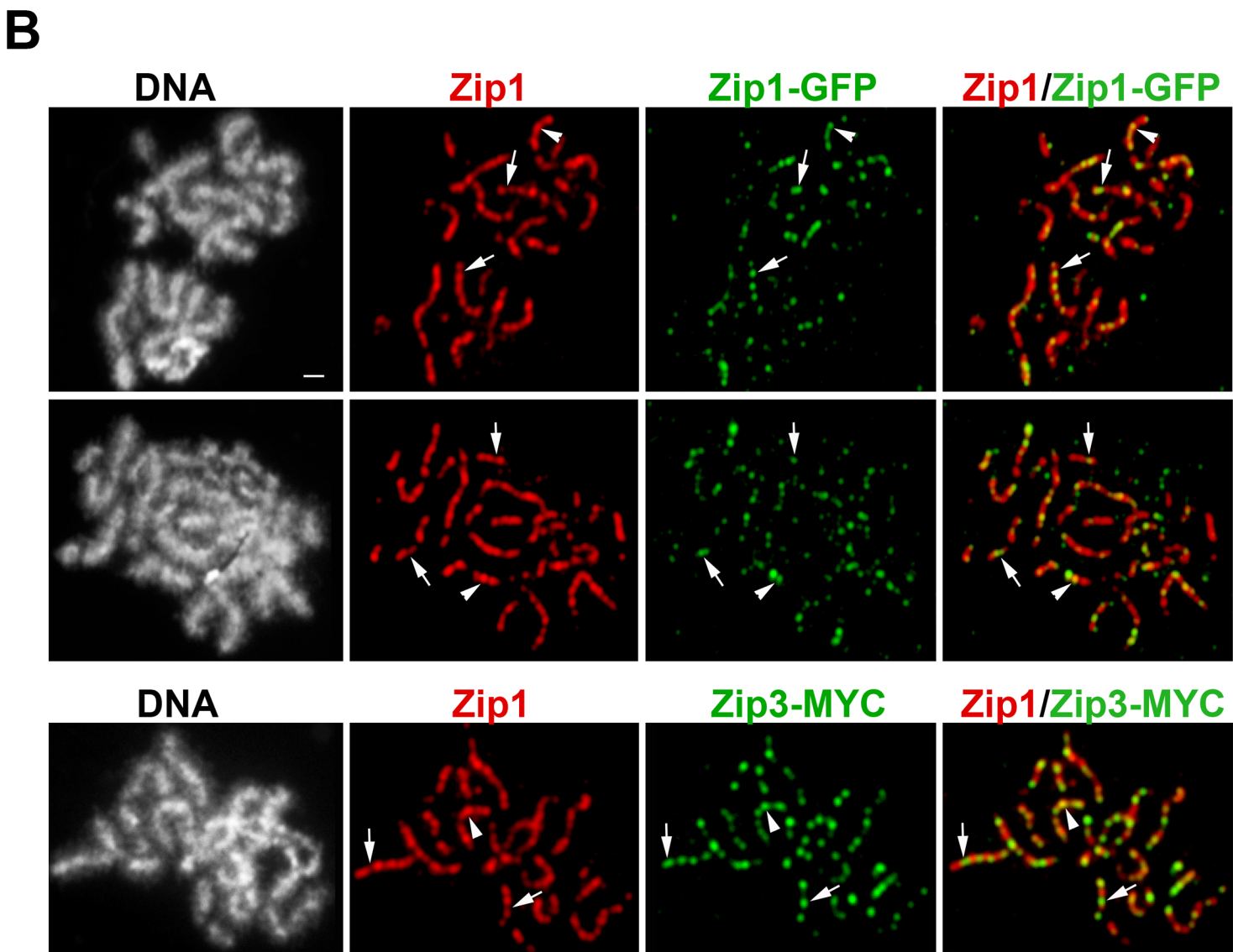

Supplement: Figure S3 — Zip1-GFP incorporates into full-length SC both at sites of high and sites of low Zip1 abundance. Graph in (A) gives the number of discrete Zip1-GFP incorporation events (focus or short (0.35–0.5 µm) stretch) or Zip3-MYC foci that co-localized with an area of high Zip1 abundance (“Zip1 Peak”), low Zip1 abundance (“Zip1 Valley”), or at the terminus of a synapsed chromosome (“Zip1 Termini”) in K48. Zip1-GFP incorporation events or Zip3-MYC foci that could not be unambiguously assigned to a particular feature of the SC were grouped in the “Ambiguous” category. Images in (B) show examples of Zip1-GFP events (green, top two rows) or Zip3-MYC foci (green, bottom row) in Zip1 (red) valleys (arrows) or peaks (arrowheads). DNA for these nuclei is shown in white (at left). Scale, 1 µm. (PDF) [file pgen.1002993.s003.pdf]

Figure S4

A

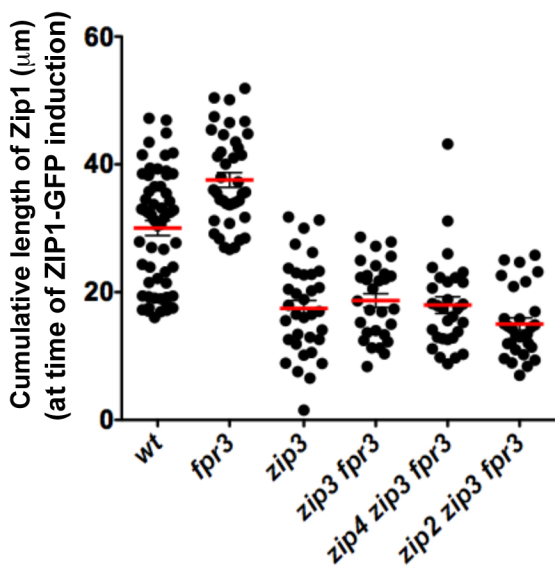

B

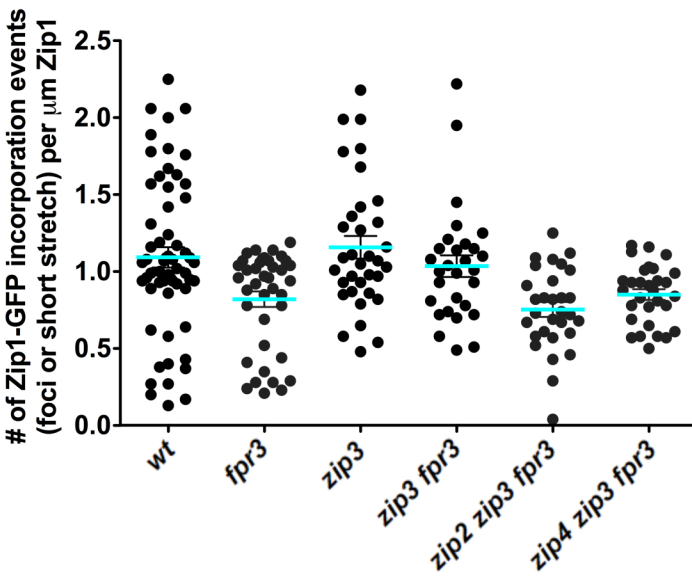

Supplement: Figure S4 — Zip1-GFP incorporation into full length SC may be reduced in situations where the crossover: Zip1 length ratio is predicted to be low. Scatterplots in (A) show the extent of Zip1 SC present in nuclei, upon ZIP1-GFP induction (at “start”), for the various strains involved in the incorporation experiment shown in Figure 5. Scatterplot in (B) depicts the number of discrete Zip1-GFP incorporation events (either a focus or a Zip1-GFP stretch) per cumulative length of Zip1, after a 45-minute induction of ZIP1-GFP expression. Circles indicate Zip1-GFP incorporation events per cumulative length of Zip1 for individual nuclei. Horizontal and error bars indicate mean and standard error of the mean for each column. The average number of Zip1-GFP incorporation events per µm of Zip1 is significantly reduced (compared to wild type, Mann-Whitney test) for the following strains: zip2 zip3 fpr3 (two-tailed P = 0.0005) and zip4 zip3 fpr3 (two-tailed P = 0.0032). The fpr3 strain also exhibited a significant difference from wild type (two-tailed P = 0.0113), likely due to the longer cumulative lengths of Zip1 exhibited by this single mutant (see A). (PDF) [file pgen.1002993.s004.pdf]

Figure S5

haploid a/ $\alpha$

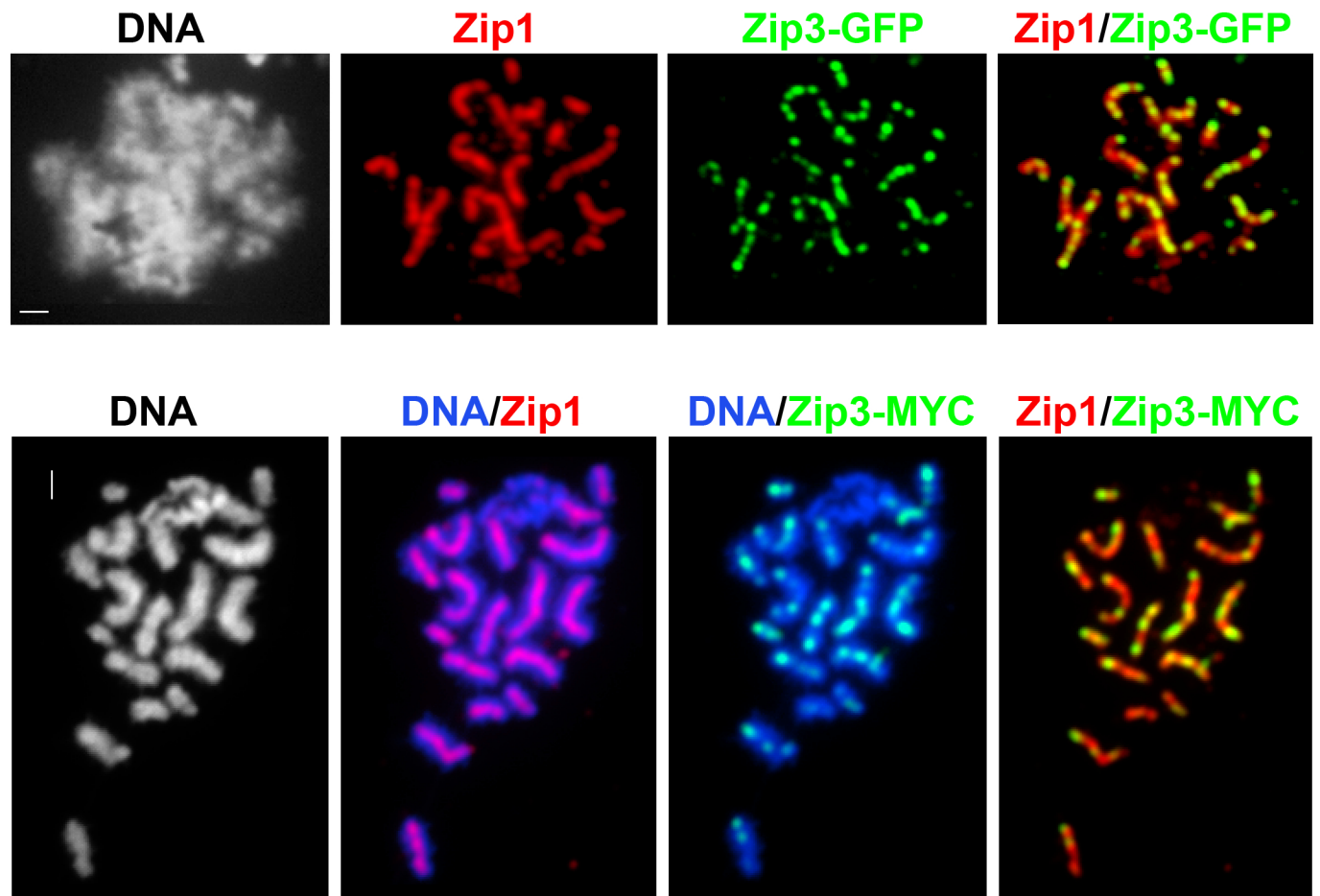

Supplement: Figure S5 — Zip3 foci decorate Zip1 stretches in haploid meiotic cells. Meiotic chromosomes (white) from haploid MATa/MATα cells carrying Zip3-GFP (AM2632, top row) or Zip3-MYC (K150, bottom row) were sporulated for 26 hours, and then surface spread on glass slides. Zip1 (red) and Zip3-GFP or Zip3-MYC (green) are depicted in single channels and as a merged image. Scale, 1 µm. (PDF) [file pgen.1002993.s005.pdf]

Figure S6

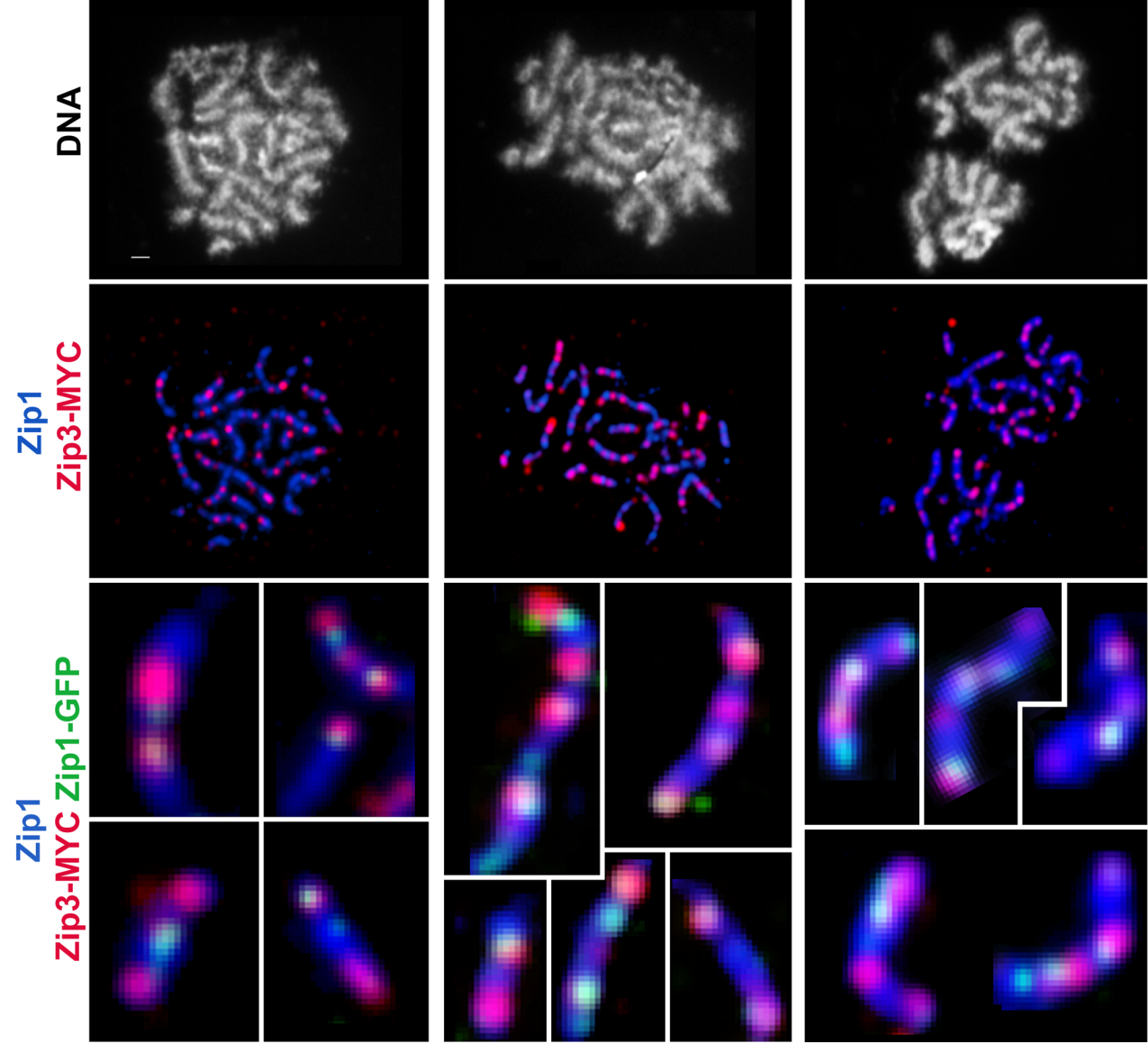

Supplement: Figure S6 — Sites of initial Zip1 entry into full-length SC localize near Zip3 foci. (A) Three surface-spread meiotic nuclei (DNA for each nucleus is shown in white, top row) from Zip3-MYC strains (K48) after a short induction of ZIP1-GFP expression (45 minutes). Zip1 staining (second row, blue) shows full length SC decorated by Zip3-MYC (red) foci. Bottom row displays several zoomed Zip1 SC stretches (blue) from each nucleus (4–5 per nucleus), which are a subset of the 69 stretches used in our statistical analysis of post-synapsis Zip1-GFP distribution relative to Zip3-MYC. Both Zip3-MYC (red) and post-synapsis Zip1-GFP incorporation events (green) are shown in zoomed images. Scale, 1 µm. (PDF) [file pgen.1002993.s006.pdf]
