# Supplementary material for: Full-Length Synaptonemal Complex Grows Continuously during Meiotic Prophase in Budding Yeast
Source: PLoS Genet. 2012 Oct 11;8(10):e1002993. doi: 10.1371/journal.pgen.1002993 (PMC3469433; doi:10.1371/journal.pgen.1002993)
Supplement: Table S1 — Strains used in this study. (PDF) [file pgen.1002993.s007.pdf]

Table S1.

NAME	GENOTYPE
**K39**	*MATα TRP1-P_{GAL1}-ZIP1-GFP ura3-1 ndt80Δ::LEU2*<br>*MATa ZIP1 ura3::P_{GPD1}-GAL4(848).ER::URA3 ndt80Δ::LEU2*<br><br>*CTF19-c-MYC::kanMX4 his4-260,519 leu2-3,112 trp1-289 ade2-1 thr1-4 lys2ΔNhe*<br>*CTF19-c-MYC::kanMX4 his4-260,519 leu2-3,112 trp1-289 ade2-1 thr1-4 lys2ΔNhe*
**K29**	K39 *TRP1-P_{GAL1}-ZIP1/zip1-4LA lys2ΔNhe/LYS2*
**K30**	K39 *TRP1-P_{GAL1}-ZIP1-GFP/zip1-4LA lys2ΔNhe/LYS2*
**K40**	K39 *TRP1-P_{GAL1}-ZIP1/TRP1-P_{GAL1}-ZIP1*
**K48**	K39 *ZIP3/ ZIP3-c-MYC*
**K59**	K39 *zip3Δ::LYS2/zip3Δ::LYS2*
**K62**	K40 *CTF19/CTF19 NDT80/NDT80*
**K83**	K39 *zip3Δ::LYS2/zip3Δ::LYS2 fpr3Δ::kanMX4/fpr3Δ::kanMX4*
**K84**	K39 *fpr3Δ::kanMX4/fpr3Δ::kanMX4*
**K91**	*K39 zip4Δ::hphMX4/zip4Δ::hphMX4 zip3Δ::LYS2/zip3Δ::LYS2*<br>*fpr3Δ::kanMX4/fpr3Δ::kanMX4*
**K92**	K39 *zip2Δ::hphMX4/zip2Δ::hphMX4 zip3Δ::LYS2/zip3Δ::LYS2*<br>*fpr3Δ::kanMX4/fpr3Δ::kanMX4*
**K117**	K39 *pch2Δ::hphMX4/pch2Δ::hphMX4 CTF19/CTF19*
**AM2560**	K39 *pph3Δ::kanMX4/pph3Δ::kanMX4 ndt80Δ::hphMX4/ndt80Δ::hphMX4*
**K139**<br>**haploid**<br>**a/α**	***MATα** TRP1-P_{GAL1}-ZIP1-GFP ura3::P_{GPD1}-GAL4(848).ER::URA3 ndt80Δ::hphMX4*<br>*CTF19-c-MYC::kanMX4 **MATa**@THR1 his4-260,519 leu2-3,112 trp1-289 ade2-1*<br>*lys2ΔNhe* R1589 (pUC18 *lacZ' CEN4 ARS1 LEU2 ZIP1*)
**AM2632**<br>**haploid**<br>**a/α**	***MATα** Zip3-GFP::URA3 Ctf19-c-MYC::kanMX4 ndt80Δ::hphMX4 **MATa**@THR1*<br>*his4-260,519 leu2-3,112 trp1-289 ade2-1 lys2ΔNhe* R1589 (pUC18 *lacZ' CEN4 ARS1*<br>*LEU2 ZIP1*)
**K150**<br>**haploid**<br>**a/α**	K139 *ZIP3-c-MYC CTF19*
**SM170**	*MATα TRP1-P_{GAL1}-ZIP1 ura3-1 ndt80Δ::LEU2*<br>*MATa ZIP1-YFP ura3::P_{GPD1}-GAL4(848).ER::URA3 ndt80Δ::LEU2*<br><br>*his4-260,519 leu2-3,112 trp1-289 ade2-1 thr1-4 lys2ΔNhe*<br>*his4-260,519 leu2-3,112 trp1-289 ade2-1 thr1-4 lys2ΔNhe*
**SM176**	SM170 *ZIP1-YFP/ZIP1-YFP*
**SM224**	SM176 *ndt80Δ::hphMX4/ndt80Δ::hphMX4 leu2::ZIP1-YFP::LEU2/leu2-3,112*
**SM232**	SM224 *leu2::ZIP1-YFP::LEU2/ leu2::ZIP1-YFP::LEU2*
**SM240**	SM170 *TRP1-P_{GAL1}-ZIP1-YFP/TRP1-P_{GAL1}-ZIP1-YFP ndt80Δ::hphMX4/ndt80Δ::hphMX4*<br>*leu2::ZIP1-YFP::LEU2/leu2-3,112*
**SM248**	SM170 *TRP1-P_{GAL1}-ZIP1/TRP1-P_{GAL1}-ZIP1 ndt80Δ::hphMX4/ndt80Δ::hphMX4*<br>*leu2::ZIP1-YFP::LEU2/leu2-3,112*

**AM2614**	*MAT**a**/**α** zip1Δ::natMX4/zip1Δ::natMX4 leu2::ZIP1-YFP::LEU2/leu2::ZIP1-YFP::LEU2*
**AM2648**	SM170 *ZIP1-YFP /zip1Δ::natMX4 NDT80/NDT80*
**AM2650**	AM2648 *ZIP1-YFP/ ZIP1-YFP*
**AM2652**	AM2650 *leu2::ZIP1-YFP::LEU2/leu2-3,112*
**AM2654**	AM2650 *leu2::ZIP1-YFP::LEU2/ leu2::ZIP1-YFP::LEU2*
**BR1919**	*MAT**α** ura3-1 his4-260,519 leu2-3,112 trp1-289 ade2-1 thr1-4*<br>*MAT**a** ura3-1 his4-260,519 leu2-3,112 trp1-289 ade2-1 thr1-4*
