# Supplementary material for: Full-Length Synaptonemal Complex Grows Continuously during Meiotic Prophase in Budding Yeast
Source: PLoS Genet. 2012 Oct 11;8(10):e1002993. doi: 10.1371/journal.pgen.1002993 (PMC3469433; doi:10.1371/journal.pgen.1002993)
Supplement: Table S2 — Viability of spores produced by diploids carrying one to six copies of ZIP1. The far right column shows the overall spore viability for each strain. Displayed in each “Distribution of tetrad types” column is the frequency of tetrads containing four viable spores (4-sv), three viable spores (3-sv), two viable spores (2-sv), one viable spore (1-sv) or no viable spores (0-sv). Strain genotypes are listed in Table S1. (PDF) [file pgen.1002993.s008.pdf]

Table S2

Strain	Tetrads dissected	Distribution of tetrad types (%)					Spore viability (%)
		4-sv	3-sv	2-sv	1-sv	0-sv	
ZIP1-1COPY (AM2648)	52	37	6	7	2	0	88
ZIP1-2COPY (AM2650)	52	44	7	1	0	0	95
ZIP1-4COPY (AM2652)	52	44	6	2	0	0	95
ZIP1-6COPY (AM2654)	52	44	5	3	0	0	95

Viability of spores produced by diploids carrying one to six copies of *ZIP1*. The far right column shows the overall spore viability for each strain. Displayed in the "Distribution of tetrad types" columns are the frequency of tetrads containing four viable spores (4-sv), three viable spores (3-sv), two viable spores (2-sv), one viable spore (1-sv) or no viable spores (0-sv). Strain genotypes are listed in Supplementary Table S1.
